# High affinity anchoring of the decoration protein pb10 onto the bacteriophage T5 capsid

**DOI:** 10.1038/srep41662

**Published:** 2017-02-06

**Authors:** Emeline Vernhes, Madalena Renouard, Bernard Gilquin, Philippe Cuniasse, Dominique Durand, Patrick England, Sylviane Hoos, Alexis Huet, James F. Conway, Anatoly Glukhov, Vladimir Ksenzenko, Eric Jacquet, Naïma Nhiri, Sophie Zinn-Justin, Pascale Boulanger

**Affiliations:** 1 Institute for Integrative Biology of the Cell (I2BC), CEA, CNRS, Univ Paris-Sud, Université Paris-Saclay, Gif-sur-Yvette cedex, France; 2 Institut Pasteur, Biophysique Moléculaire, Citech, UMR 3528, Paris, France; 3 Department of Structural Biology, University of Pittsburgh School of Medicine, Pittsburgh, Pennsylvania, USA; 4 Institute of Protein Research, Russian Academy of Sciences, Pushchino, Russia; 5 Institut de Chimie des Substances Naturelles, Univ Paris-Sud, Université Paris-Saclay, CNRS UPR 2301, Gif-sur-Yvette, France

## Abstract

Bacteriophage capsids constitute icosahedral shells of exceptional stability that protect the viral genome. Many capsids display on their surface decoration proteins whose structure and function remain largely unknown. The decoration protein pb10 of phage T5 binds at the centre of the 120 hexamers formed by the major capsid protein. Here we determined the 3D structure of pb10 and investigated its capsid-binding properties using NMR, SAXS, cryoEM and SPR. Pb10 consists of an α-helical capsid-binding domain and an Ig-like domain exposed to the solvent. It binds to the T5 capsid with a remarkably high affinity and its binding kinetics is characterized by a very slow dissociation rate. We propose that the conformational exchange events observed in the capsid-binding domain enable rearrangements upon binding that contribute to the quasi-irreversibility of the pb10-capsid interaction. Moreover we show that pb10 binding is a highly cooperative process, which favours immediate rebinding of newly dissociated pb10 to the 120 hexamers of the capsid protein. In extreme conditions, pb10 protects the phage from releasing its genome. We conclude that pb10 may function to reinforce the capsid thus favouring phage survival in harsh environments.

Bacteriophages, viruses that infect bacteria, are highly stable particles that protect the viral genome in a broad range of environnemental conditions while also recognizing and infecting their hosts. The vast majority of known bacterial viruses are tailed bacteriophages that consist of an isometric or prolate icosahedral capsid containing a double-stranded DNA molecule and a tail that attaches to the dodecameric portal complex located at a unique capsid vertex. Proteins of the tail tip bind a specific bacterial receptor, triggering release of the DNA from the capsid through the tail tube and its delivery into the host cell.

The icosahedral lattice of phage capsids is formed of identical protein subunits arranged in pentamers at the vertices and hexamers on the faces, through quasi-equivalent interactions^1^. In many phages, auxiliary proteins decorate the outer surface of the capsid shell. While these auxiliary proteins are dispensable for capsid assembly, some of them provide an evolutionary advantage to the phage particle. The so-called cementing proteins have a trimeric organization and clamp together three hexamers at local 3-fold axes thus increasing phage capsid stability, like proteins gpD of phage λ^2^ and Soc of phage T4^3^. A second family of accessory proteins includes decoration proteins that bind to the centre of hexamers. Well-known examples are the monomeric Hoc protein of phage T4^4^, the trimeric collagen-like protein gp12 of phage SPP1^5^ and the dimeric Psu protein of phage P4^6^. Hoc and gp12 have no effect on capsid stability, and their function remains elusive. In contrast, Psu has a well-established role in infection and in P4 particle stability. It acts as a polarity suppressor and prevents DNA exit by covering the holes located in the centre of the P4 capsid hexamers^6^,^7^,^8^. Hoc possesses three N-terminal immunoglobulin-like domains and a C-terminal fragment rich in α-helices that is likely to be involved in T4 capsid binding^9^. Gp12 exhibits an N-terminal collagen-like motif and a C-terminal fragment rich in α-helices. Psu folds into an α-helical knotted dimer, its C-terminal helix α7 likely being involved in capsid binding. However, structural details on the interface between these auxiliary proteins and the capsid proteins are still lacking.

Here we investigate the properties of pb10, the decoration protein of bacteriophage T5 capsid. Basic principles of T5 capsid assembly and maturation have been established^10^,^11^ and follow the phage HK97 paradigm^12^. The first procapsid form, prohead I, is built from 775 copies of the head protein pb8 arranged into 11 pentamers and 120 hexamers, as well as a dodecamer of the portal protein pb7 located at the 12th vertex. The N-terminal scaffolding domain of the head protein subunits is cleaved off by the protease pb11, converting prohead I into empty prohead II. Packaging of the T5 genome into prohead II is driven through the portal gate by the terminase in an ATP-dependent reaction^13^. This process triggers the irreversible expansion of the prohead lattice resulting from the cooperative rearrangement of the head protein subunits^14^. After the termination of DNA packaging, the head completion protein p144 seals the portal vertex and forms the docking site for attachment of the pre-assembled tail. The DNA-filled capsid is decorated with 120 copies of the 17.3 kDa protein pb10, which binds as a monomer to the centre of each hexamer^15^. Pb10 is an accessory protein, which is dispensable for infection under most laboratory conditions. No structural or functional data are available for pb10, however analysis of its sequence unveils a putative C-terminal immunoglobulin-like (Ig-like) domain^16^.

Compared to other phages, T5 is an attractive system to study the mechanism of capsid decoration because it is possible to purify empty T5 capsids and trigger their expansion *in vitro*^14^, which facilitates the analysis of pb10 binding. In this report we present the 3D solution structure of pb10, supplemented with an analysis of the binding properties of this decoration protein using nuclear magnetic resonance spectroscopy (NMR), small angle X-ray scattering (SAXS), cryo electron microscopy (cryoEM) and surface plasmon resonance (SPR). These data were combined to discuss the conformation of pb10 bound to the assembled capsid. Finally, we report experiments supporting a role for pb10 in preventing DNA release in extreme conditions.

## Results

### Pb10 consists of two domains showing autonomous structure and dynamic

**Figure 1:**
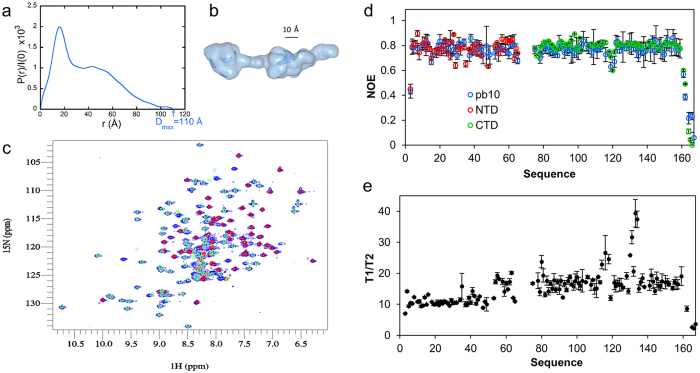
pb10 is a two-domain protein. (**a**) Distance distribution function P(r) derived from pb10 SAXS curve (see Supplementary Fig. S1) (radius of gyration: 30.0 Å; maximum extension of the protein: 110 Å). (**b**) Most typical GASBOR model of pb10. (**c**) Overlay of the ^1^H-^15^N HSQC spectra of the full-length pb10 in blue, the N-terminal domain in red and the C-terminal domain in green. (**d**) ^1^H → ^15^N NOE of the full-length pb10 in blue, the N-terminal domain in red and the C-terminal domain in green. Error bars correspond to standard deviations of four and two repeated NOE experiments for the full-length pb10 and the separate domains respectively. (**e**) Relaxation times of the individual residues in the full-length pb10. The average T1/T2 is 12.0 for the NTD and 17.2 for the CTD.

The T5 capsid decoration protein is 164 amino acids in length and includes a poly-proline motif PPAPPAPP located in the middle of the sequence and adjacent to the C-terminal domain that is predicted to be Ig-like (Supplementary Fig. S1). Chromatographic (SEC-MALLS) analysis of pb10 fused to a C-terminal histidine tag showed that the protein elutes as a monomer in a single peak corresponding to 17 kDa (Supplementary Fig. S1). The structure of pb10 in solution was investigated by SAXS. The pair distribution function P(r) derived from the scattering data (Fig. 1a) exhibits a sharp maximum at small distances followed by a regular decrease out to large distances characteristic of an elongated particle. Models derived from the SAXS data using GASBOR^17^ (Supplementary Fig. S1) consistently exhibit two domains separated by a short and straight linker (Fig. 1b). Each lobe contains about the same number (75) of pseudo-residues. The elongated domain has an Ig-like shape suggesting it is the C-terminal domain of pb10.

Based on these observations, we designed constructs to produce the two domains of pb10 separately. The N-terminal domain (NTD) construct - residues 1 to 77 - includes the poly-proline region and the first five residues of the C-terminal domain (CTD), while the CTD construct only includes residues 72 to 164 that constitute the putative Ig-like domain. We used heteronuclear NMR to further investigate the two-domain organization of pb10. We first compared the ^1^H-^15^N HSQC (heteronuclear single quantum coherence) spectra of the separate domains and full-length protein (Fig. 1c). The spectrum of each domain superimpose well with the full-length pb10 spectrum, suggesting that the two domains fold independently from each other and exhibit the same three-dimensional structure alone and within the pb10 protein. Moreover, NMR {^1^H-^15^N} NOE (nuclear Overhauser effect) experiments recorded on both the separate domains and the full-length pb10 confirmed that the two domains are well-structured (most {^1^H-^15^N} NOE values are above 0.6) and indicated that they exhibit similar short (picosecond to nanosecond) time scale dynamics when isolated or in the full length pb10 (Fig. 1d). NMR ^15^N relaxation times T_1_ and T_2_ were measured on the full-length pb10 (Fig. 1e) to determine how constrained the motions of the two domains are relative to each other. The ratio T_1_/T_2_ that reflects global motion differs between the two domains: the NTD has an average T_1_/T_2_ value of 12.0 whereas the CTD has a value of 17.2. This indicates that the two domains have different overall rotational correlation times, demonstrating that the linker allows independent motion of the CTD with respect to the NTD.

### The N-terminal domain adopts an α-helical fold in solution

**Figure 2:**
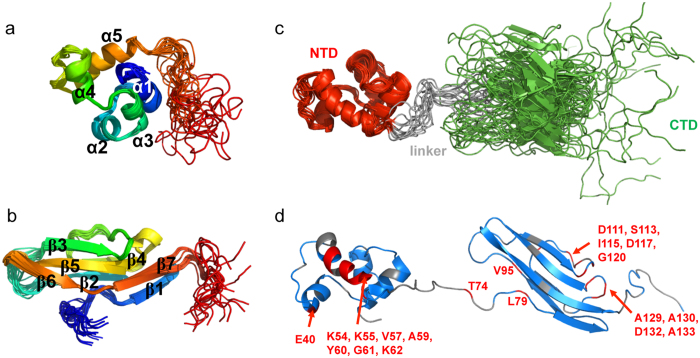
Three dimensional solution structure of free pb10 protein. (**a**,**b**) Superposition of 20 backbone structures of the pb10 (**a**) NTD and (**b**) CTD. Structures are coloured from blue (N-terminus) to red (C-terminus). The 5 α-helices and 7 β-sheets are annotated. (**c**) Superposition of 15 backbone structures of the full length pb10, all consistent with the NMR and SAXS data (see Supplementary Table S2 and Fig. S4). (**d**) Spatial distribution of the pb10 residues exhibiting significant conformational exchange, as revealed by a Modelfree analysis of pb10 ^15^N relaxation data (see Supplementary Fig. S5). These residues are coloured in red, residues that are not affected by conformational exchange are in blue, whereas residues for which no relaxation data could be measured are in grey. Most of the residues affected by conformational exchange are located in NTD helix α5 and CTD loops β3β4 and β4β5.

The solution structures of the two separate domains were solved by NMR. The NTD comprises five α-helices: α1 (Y4-F11), α2 (S18-A23), α3 (V25-Y30), α4 (Y34-L41) and α5 (N49-Y60) (Fig. 2a and Supplementary Table S1). Sixteen buried residues interact to form the hydrophobic core of NTD: Y4, L7, I10, F11 (α1), L15, P16 (loop α1α2), I20, F21 (α2), Y30 (α3), V31 (loop α3α4), L37, L41 (α4), V52, W60 (α5), F56 and Y60. The solvent-accessible surface of this domain comprises 29 residues and in particular 4 negatively charged (E13, E17, E40, E58), 9 positively charged (K14, K29, R38, R39, R50, K51, K54, K55, K62) and 4 hydrophobic residues (excluding alanines; F22, V25, F36, L43). The Dali server^18^ was used to search for similar 3D structures, but no protein fragment with a structurally related fold was identified that also exhibited related biological functions.

### The C-terminal domain adopts an immunoglobulin fold in solution

The CTD comprises seven β-strands: β1 (S82-E86), β2 (L91-S94), β3 (T105-K110), β4 (T122-K125), β5 (G134-D142), β6 (Q145-S150) and β7 (C153-N158) (Fig. 2b and Supplementary Table S1). These β-strands are assembled into two anti-parallel β-sheets. Strands β1, β3, β5, β6 and β7 form the first and largest β-sheet whereas strands β2 and β4 form the second β-sheet. A search for structurally similar proteins possessing using the Dali server identified many proteins containing an Ig-like fold, including immunoglobulin receptors, fibroblast growth factor receptors and the T-cell surface glycoprotein CD4. Of note, pb10 CTD resembles the N-terminal Ig-like domain of the decoration protein Hoc from the T4-like phage RB49 (PDB entry 3SHS, RMSD = 3.1 Å) as shown on Supplementary Fig. S2.

### Full length pb10 3D structure exhibits a well-conserved surface on its N-terminal domain

Full-length pb10 models were generated using a subset of the NMR derived restraints obtained on the separate N- and C-terminal domains and SAXS data obtained on the whole pb10. To build the NMR dataset, we selected restraints involving residues showing no significant ^15^N and ^1^H chemical shift changes between the separate domains and the full form of pb10 (Supplementary Fig. S3). The resulting dataset contained 2490 NOE distance restraints and 214 dihedral angle restraints. Figure 2c shows 15 pb10 models calculated using the XPLOR-NIH software^19^ and consistent with the NMR and SAXS data (Supplementary Table S2 and Fig. S4). Examination of these 15 conformations shows that the relative positioning of the NTD and CTD is restricted. However, in agreement with the NMR T_1_/T_2_ relaxation data, a residual mobility in this relative positioning is suggested by our structure calculations (Fig. 2c). Further analysis of the full-length pb10 ^15^N relaxation data using the Modelfree software^20^ revealed that 16 residues are affected by a local conformational exchange process on a microsecond to millisecond timescale (Supplementary Fig. S5). Interestingly, these residues cluster within three sites: NTD helix α5, CTD loop β3 β4 and CTD loop β4β5 (Fig. 2d). The fact that most helix α5 residues exhibit conformational exchange suggests that positioning of this helix within the NTD is dynamic.

**Figure 3:**
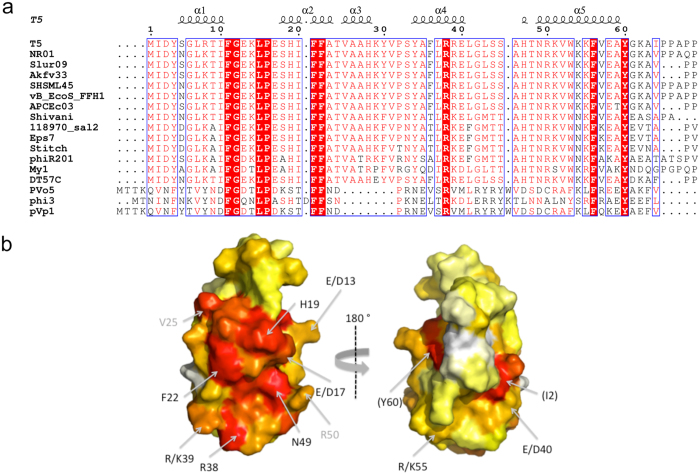
Conservation of the NTD within 14 closely related and 3 more distant decoration proteins from T5-like phages. The 14 closely related decoration proteins exhibit two or three domains, the NTD being particularly well conserved (see Supplementary Fig. S1). The more distant decoration proteins of the T5-like vibrio-phages PVo5, phi3 and pVp1(GenBank Accession Numbers KT345706, NC_028895 and JQ340389 respectively) possess a unique α-helical domain that shares the sequence motif F-G-(X)_2_-L-P-(X)_4-5_-F-F with pb10 NTD. The 17 α-helical domain sequences were aligned using MAFFT through the Consurf webserver^21^. The alignment picture was generated using ESPript 3.0^46^ and is displayed in (**a**). Secondary structure elements observed within T5 pb10 NTD are displayed above the sequences. Residues strictly conserved in all phage protein sequences are displayed on a red background. Residues conserved at more than 70% are displayed in red on a white background. Non conserved residues are displayed in black. Projection of the Consurf conservation scores onto pb10 NTD structure is shown in (**b**). The surface of pb10 NTD is coloured from red (highest conservation scores) to white (lowest conservation scores). Residues conserved in all phages are annotated in black whereas residues only conserved in the closely related phages are annotated in grey. Residues that are less than 30% solvent exposed but still visible and conserved are annotated with brackets.

In order to determine if the structural organization observed for pb10 is shared by the decoration proteins of T5-like phages, we aligned the sequences of the decoration proteins of 14 closely related phages infecting diverse enterobacteria (Supplementary Fig. S1). Most of them show a two-domain organization. The sequences of their NTD are highly conserved (43% identity in all 14 sequences), whereas the sequences of their CTD are highly divergent (4% identity). Interestingly, the decoration proteins of the more distant T5-like vibrio-phages PVo5, pVp-1 and phi3 are only constituted of a single α-helical domain, which is about 14% identical to T5 NTD and shares the sequence motif F-G-(X)_2_-L-P-(X)_4-5_-F-F with the region α1-α2 of T5 NTD (Fig. 3a). Assuming that these 3 additional decoration proteins fold as T5 NTD, we used the Consurf webserver^21^ and projected conservation scores onto the 3D structure of T5 NTD (Fig. 3b). We thus revealed that a specific surface centre on helix α2 is well conserved in the NTD of the decoration protein of T5-like phages, including closely related but also more distant vibrio-phages.

### The N-terminal domain of pb10 binds to phage T5 capsids

**Figure 4:**
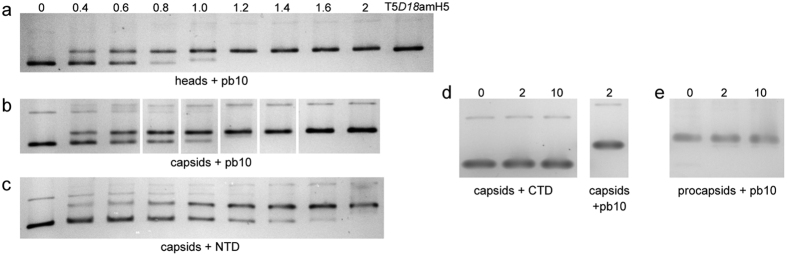
Binding assays with purified pb10 and separate domains. Different forms of T5 capsids were assayed for binding of pb10, NTD or CTD and analysed by native agarose gel electrophoresis. The concentration of hexamers was fixed at 150 nM and that of pb10, NTD and CTD varied between 60 and 300 nM. The [pb10]/[hexamer] molar ratio was calculated as described under experimental procedures and indicated above the gel. (**a**) Heads (DNA-filled capsids) incubated with pb10. The last well shows the position of native decorated DNA-filled capsids produced by T5*D18*amH5 mutant. (**b**–**d**) Expanded empty capsids incubated with pb10, NTD or CTD respectively. (**e**) Procapsid (empty non-expanded capsids) incubated with pb10.

*In vitro* decoration of T5 capsid with purified pb10 was analysed by electrophoresis on native agarose gels, which is a convenient way to resolve different capsid forms according to their shape and exposed charges. We mixed DNA-filled heads lacking pb10 or empty expanded capsids with increasing concentrations of full-length pb10 or separate domains. Addition of pb10 to the capsid particles modified their electrophoretic mobility, shifting the band of pb10-free heads (Fig. 4a) or expanded capsids (Fig. 4b) to a slower migrating band corresponding to fully decorated particles. At a [pb10]/[hexamer] molar ratio of 0.4, most heads or expanded capsids lacked pb10 but a minority of particles were already saturated, while at a ratio of 1.2, almost all particles were totally decorated. This behaviour suggests that binding of pb10 to the capsids is cooperative, which is analysed below. The NTD of pb10 binds to the capsids leading to full decoration at a [NTD]/[hexamer] ratio of 1.6 (Fig. 4c). In contrast, the CTD does not bind to the capsid even when added at a tenfold molar ratio (Fig. 4d). These data revealed that the well-conserved NTD constitutes the binding domain of pb10, while the CTD likely constitutes an external domain exposed to the solvent. No shift of the non-expanded procapsid band was observed in the presence of pb10 (Fig. 4e). This shows that the structural reorganization of the capsid protein subunits that takes place upon expansion creates the binding sites for the decoration protein, as observed in other phages^2^.

### Pb10 binds to T5 capsids with high affinity and high cooperativity

**Figure 5:**
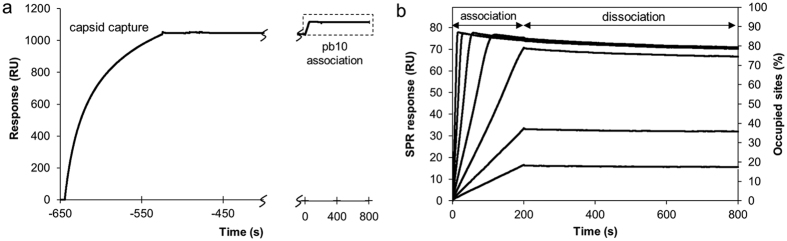
Pb10 binds to T5 capsids with a picomolar affinity. (**a**) SPR real-time profile (resonance units, RU) corresponding to the capture of empty expanded capsid (120 seconds) followed by pb10 association (200 s) and dissociation (600 s) at the saturating concentration of 5 nM. The dotted square corresponds to the area presented in (**b**). (**b**) Association and dissociation of pb10 at increasing concentrations (0.312, 0.625, 1.25, 2.5, 5, 10, 20 nM) on empty expanded capsids.

To further characterize the interaction between pb10 and the T5 capsid, the kinetics of pb10 binding was investigated by SPR. We captured expanded capsids non-covalently on a SPR sensor chip through anti-capsid antibodies. Only marginal capsid dissociation was observed over time (Fig. 5a). Association and dissociation of pb10 were monitored at concentrations of pb10 ranging from 0.3 to 20 nM (Fig. 5b). The full decoration of the 26 MDa T5 empty capsid by 120 copies of the 18.3 kDa pb10-His leads to a calculated mass increase of 8.4%. Our data showed a maximum mass increase of about 8.0% suggesting almost full decoration. Association of pb10 on T5 capsids is fast, while dissociation is remarkably slow. Indeed, only 7% of signal decrease was observed over 700 s of dissociation. The dissociation rate constant k_off_ of pb10 from the T5 capsid could not be determined accurately as it was close to that of the capsid/anti-capsid antibody interaction. Averaging over 8 experiments led to a k_off_ value of about 2 10^−4^ s^−1^. Setting this value as a constant, the dissociation equilibrium constant (K_D_) was calculated as 1.1 10^−12^ M (Table 1). The NTD binding kinetics did not reveal any significant difference compared to the full-length pb10 binding kinetics (Table 1). Thus, the CTD does not appear to influence the binding properties of the NTD, which is in agreement with the structural data showing that both domains fold independently.

At sub-saturating concentrations of pb10, partially decorated particles with an intermediate electrophoretic mobility between saturated and undecorated capsids were poorly detected on agarose gels. This suggests that binding of pb10 on its 120 hexameric sites is cooperative. We tried to observe partially decorated capsids in SPR experiments by mixing pb10 with a large excess of binding sites. To this end, we incubated expanded capsids with sub-saturating concentrations of His-tagged pb10, and we captured these decorated capsids on a Ni^2+^-NTA sensor chip: only capsids having incorporated at least one pb10 protein were thus retained. We then injected untagged pb10 over these capsids. When the capsids were initially incubated with a low molar ratio of 6.8 × 10^−3^ pb10 per hexamer, association of untagged pb10 could be observed, showing that capsids were not fully decorated (Supplementary Fig. S6). Alternatively, when the capsids were incubated with a 10-fold higher ratio of pb10 (6.8 × 10^−2^ per hexamer), no further binding of untagged pb10 could be detected, showing that capsids were already fully decorated. These results demonstrate that partially decorated capsids do exist. However, as suggested by the capsid profiles on agarose gels, this population of capsids is very scarce and can only be seen when binding sites are in large excess over the number of available pb10 molecules. The decoration process is thus highly cooperative. The association of a few copies of pb10 is probably enough to trigger conformational changes over the whole capsid surface thus promoting efficient saturation by pb10.

### Density corresponding to the NTD is observed at the centre of T5 capsid hexamers

**Figure 6:**
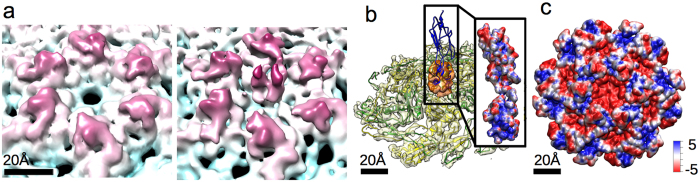
Surface views of hexamers from 3D reconstructions of capsids imaged by from cryo-EM. (**a**) Radially coloured close view of one hexamer of the capsid without pb10 (left) and with pb10 (right). (**b**) Fitting of the model of pb8 and pb10 in the density map of the capsid with pb10. (**c**) +/−5 kT/e electrostatic calculation of the model of pb10 and pb8 using APBS and 150 mM of mobile ions.

To explore the nature of the interaction between pb10 and the major capsid protein pb8, we first searched for the position of pb10 on capsid structures determined by cryo-electron microscopy and image reconstruction. Two density regions corresponding to a single hexamer of the empty expanded capsid observed without and with pb10 were obtained (Fig. 6a, estimated resolution of 8 and 9 Å, respectively). Pb8 adopts the major capsid protein fold first observed in the X-ray structure of the phage HK97 capsid, and its 3D structure was modelled on this basis^10^,^15^. Six copies of this pb8 model were fit into the density of one hexamer in both cryoEM maps and the density corresponding to pb10 was identified (Fig. 6b). We did not observe any additional density inside the capsid in the map of the pb10-bound capsid, showing that pb10 is located at the outer surface of the capsid. The density corresponding to pb10 observed at the centre of each pb8 hexamer has a size compatible with the NTD of pb10. It appears as an averaged low-resolution lump, likely due to the superposition of one pb10 monomer in each of 6 quasi-equivalent positions on the hexamer (Fig. 6b). Consequently it was not possible to fit the NMR NTD structure into this density. The CTD density is not distinguishable from the noise, suggesting that its position relative to the capsid is variable.

### The pb10 NTD and the capsid hexamer show oppositely-charged electrostatic surfaces

**Figure 7:**
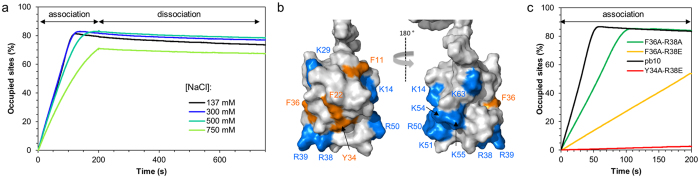
Alteration of pb10-capsid interaction by salt or mutation of solvent-exposed residues. (**a**) Association (0 to 200 s) and dissociation (200 to 800 s) of pb10 on empty expanded capsids in PBS containing 137, 300, 500 or 750 mM NaCl. (**b**) Surface representation of the NTD with mutated hydrophobic residues coloured in orange and positively charged residues in blue (same orientation as in Fig. 3b). (**c**) Association of 5 nM wild-type pb10 and several mutants with a reduced affinity on empty expanded capsids.

We calculated the electrostatic potential at the surface of both the pb8 hexamer and the pb10 models. The centre of the hexamers is formed by six copies of the A-loop of pb8 (named by homology with the equivalent loop in the HK97 capsid protein) and its surface is negatively charged due to the presence of multiple aspartate and glutamate residues in these loops (Fig. 6c and Supplementary Fig. S7). In contrast, the pb10 NTD exhibits a positive electrostatic potential (Fig. 6b). This suggests that electrostatic interactions contribute to the high-affinity binding of pb10 to the T5 capsid. We investigated these interactions by increasing the concentrations of NaCl in SPR experiments (Fig. 7a, Table 1). Fitting of the association curves clearly showed that the association rate decreases when the concentration of NaCl increases. In contrast, the very slow dissociation rate was not affected, suggesting that other mechanisms govern the locking of pb10 onto the capsid.

### Mutations in pb10 NTD do not significantly impact dissociation of the pb10-capsid complex

In order to further identify pb10 residues involved in T5 capsid binding, we designed several variants of pb10 with point mutations of exposed positively charged or aromatic residues. Lysines and arginines were replaced by either glutamic acid to reverse the charge or by alanine to suppress it, while phenylalanines and tyrosines were replaced by alanines (Table 1, Fig. 7b). Binding properties of the resulting pb10 mutants were assayed on agarose gels. A first screen was performed in order to test the role of three exposed positively charged residues (R38 in α4, R50 and K54 in α5) and four aromatic residues (the solvent exposed F22 in α2 and F36 in α4 and the more buried F11 in α1 and Y34 in α4) (Supplementary Fig. S8). Surprisingly, only F11A and F22A bound less efficiently but also only these mutants exhibited an altered circular dichroism (CD) spectrum, indicating that their α-helical structure is modified by the mutation (Supplementary Fig. S8). We then performed a much larger screen to test the impact of single, double and even quadruple mutations of pb10 on T5 capsid binding (Supplementary Fig. S8). We tested the role of the 6 remaining solvent exposed and positively charged residues (K14 in loop α1α2, K29 in α3, R39 in α4, K51, K55 in α5 and K62 at the C-terminus of NTD). However their substitution by alanine did not significantly impact capsid binding. We tested combinations of mutations: either one aromatic and one positively charged residues or two positively charged residues or even four positively charged residues (Table 1). Here again, only Y34A-R38E, F36A-R38A and F36A-R38E showed less efficient binding but also an altered CD spectrum (Supplementary Fig. S8). Their binding kinetics was estimated by SPR (Fig. 7c, Table 1). We measured reduced association constants (10 to 500-fold decreases in k_on,_ correlated to the changes seen in agarose gels) but we did not detect any changes in the dissociation rates. The changes in CD spectra and association rates were specifically triggered by the double mutations, as the corresponding single-mutants behaved as wild-type pb10 (only a slight decrease in k_on_ was observed by SPR for R38E). These results suggest that the NTD 3D structure is a determinant of the association rate, but again underlined that other mechanisms govern binding and locking of pb10 onto the capsid.

### Pb10 stabilizes the capsid and protects the phage from DNA release

**Figure 8:**
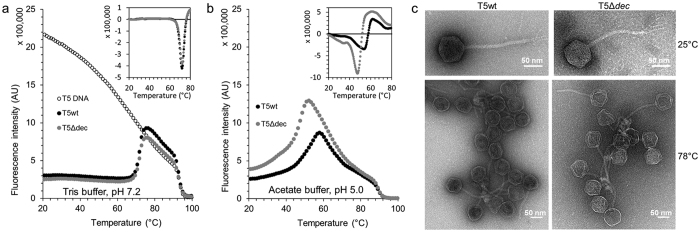
Decoration delays phage DNA exit at high temperature. (**a**,**b**) DNA release from T5wt (black) and non-decorated T5Δdec (grey) phages challenged with a linear temperature gradient (3 °C/min) and monitored by fluorescence intensity of SYBR-Gold probe in (**a**) 20 mM Tris-HCl buffer pH 7.2, 100 mM NaCl, 1 mM CaCl_2_ and 1 mM MgCl_2_ and (**b**) 20 mM sodium acetate buffer pH 5.0, 300 mM NaCl, 1 mM CaCl_2_ and 0.1 mM MgCl_2_. Only every third point is shown for clarity. Insets: opposite first derivative of the fluorescence curves calculated with QuantStudio 12 K Flex software v1.2.2. (**c**) Negative stain EM images of T5 particles with (left) and without (right) pb10 in 20 mM sodium acetate buffer pH 5.0, 100 mM NaCl, 1 mM CaCl_2_, 1 mM MgCl_2_. Top: non heated particles. Bottom: after heating at 1 °C/min up to 78 °C.

The strong association of pb10 to the capsid, together with the conservation of the N-terminal domain in all T5-like phages, suggests that the decoration protein may provide an advantage to the phage. In order to investigate the role of pb10 in capsid stability we assessed the resistance of phage particles upon heating under various physico-chemical conditions. Phages are known to release their DNA when the temperature reaches a given threshold, which depends on the structure and characteristics of the capsid lattice^22^,^23^,^24^. T5 phages with or without pb10 were challenged with a linear gradient of temperature and DNA released in the external medium was quantified using the fluorescent probe SYBR-Gold. Typical fluorescence assays performed in the usual T5 storage buffer are shown in Fig. 8a. Fluorescence intensity of SYBR-gold in the presence of DNA decreased with increasing temperature, due to the temperature-dependence of the probe fluorescence. T5 phages with or without pb10 exhibited overlapping curves with a stable low signal followed by a fluorescence increase starting from about 70 °C. The fluorescence signal reached above 75 °C was similar to that observed for identical amounts of free T5 DNA, indicating that DNA was fully released from the phage particles. The temperature of DNA exit (*T*_*ex*_) was determined from the peak visualized by plotting the fluorescence first derivative (Fig. 8a, inset). Under these standard conditions, the presence of pb10 did not influence the temperature of DNA release. We repeated these same experiments in a series of buffers characterized by different pH ranging from 8.2 to 5.0 and different concentrations of NaCl and MgCl_2_. *T*_*ex*_ decreased when the NaCl concentration increased from 0 to 300 mM. The *T*_*ex*_ decrease was more evident at acidic pH and at lower Mg^2+^ concentrations (Table 2). At 300 mM NaCl, pH 5.0 and 0.1 mM MgCl_2_, phages lacking pb10 were significantly less temperature-resistant, with *T*_*ex*_ = 47.5 ± 0.2 °C compared to 53.8 ± 0.1 °C for wild-type phages (Fig. 8b and Table 2). These differences revealed that the decoration protein enables the phage to retain its genome in critical ionic conditions. Negative stain EM images of T5 particles produced either with or without pb10 and heated at 78 °C in acetate buffer at pH 5.0 showed that capsids were neither destroyed nor disconnected from the tail tube (Fig. 8c), suggesting that the head-to-tail connector opened to release DNA. On this basis, we propose that decoration of the capsid lattice reinforces the stability of the head-to-tail connector regulating DNA exit in extreme conditions.

## Discussion

Here we present one of the first complete three-dimensional structures of a decoration protein that binds to the centre of phage capsid hexamers, solved from the combination of SAXS and NMR data. The monomeric protein pb10 of phage T5 is composed of two structural and functional domains that fold independently and are connected by a poly-proline linker. The elongated conformation of the linker, predicted from its sequence rich in alanines and prolines^25^ and observed in our structure calculations, might favour the accessibility of each domain to their partners. The α-helical NTD is more conserved and binds to the capsids. In contrast, the C-terminal Ig-like domain is exposed to the solvent and moves partially independently from the NTD, in free pb10 and in the viral particle. Ig-like domains are frequently observed on the surface of phage structural proteins^26^, like the Hoc decoration proteins of T4-like phages^9^ or the tail tube protein of phage lambda^27^. They were proposed to interact with bacterial carbohydrates. Hence, the pb10 CTD might help T5 adsorption on the host cell or could be involved in a symbiotic interaction with metazoans by attaching to mucus as proposed for the Hoc protein of phage T4^28^.

Pb10 and its NTD bind with picomolar affinity to the T5 capsid: the decoration process is quasi-irreversible. Such an affinity is, to our knowledge, the highest affinity determined for a decoration protein/capsid interaction. For example the K_D_ is 100 to 10,000 fold lower than that determined for phage L decoration protein binding to phage P22 capsids^29^ and for the Hoc protein of phage T4^30^. The dissociation rate characterizing this interaction is extremely slow, estimated around 2 × 10^−4^ s^−1^. Pb10 binds to and displays electrostatic complementarity with the centre of the capsid hexamers, however an increase in salt concentration decreases the association rate without increasing the dissociation rate of the complex. Thus, electrostatic interactions alone are not responsible for the quasi-irreversible binding of pb10. Single and double mutations in pb10 that decrease the association rate for capsids were identified, but surprisingly (1) all these mutations affect the 3D structure of pb10 and (2) none of these mutations cause a significant change in the dissociation rate of the pb10/capsid complex. First, the effect of these mutations on the association rate is correlated to the decrease in α-helical content as measured by CD (Supplementary Fig. S8): if wild-type pb10 binds T5 capsids with a k_on_ of 180 × 10^6^ M^−1^s^−1^ and has an α-helical content of 18% in its free form, R38E and F36A-R38A display k_on_ values of 61 to 84 × 10^6^ M^−1 ^s^−1^ and α-helical contents of 14%, and F36A-R38E and Y34A-R38E exhibit k_on_ values of 0.4 to 19 × 10^6^ M^−1^ s^−1^ and an α-helical content of 6%. This shows that recognition of the pb10 fold by the capsid hexamer is an important determinant of the association rate. Second, mutating four positively charged residues into alanine (R38A-R39A-R50A-K51A) or introducing double mutations that strongly perturb the NTD fold (F36A-R38E and Y34A-R38E) does not significantly modify the measured dissociation rate. These unexpected results raise the question of what mechanisms govern the dissociation rate of the pb10-capsid complex.

Pb10 binds asymmetrically at the centre of a pb8 hexamer. This binding event is cooperative: partially decorated capsids are only observed when their pb10 binding sites are in large excess over the number of available pb10 molecules. Such cooperativity strongly suggests that binding of first pb10 molecules to a T5 capsid triggers a conformational change at the level of the pb8 hexamer that is transmitted to the neighbouring hexamers to favour more pb10 binding. Thus, after dissociation of pb10 from the capsid, the newly unbound protein is immediately in close proximity to 120 capsid hexamers, and these binding sites are in a conformation favourable for pb10 binding if the capsid is already decorated by other pb10 molecules. A combination of these cooperativity and avidity processes might explain at least partially the remarkably slow dissociation rate of the complex.

The sequences of the major capsid protein A-loops forming the centre of the hexamers are highly conserved in T5-like enterobacteria phages and vibrio-phages: 50% of the loop residues are completely conserved and 75% are similar in all 17 sequences (Supplementary Fig. S7). The surfaces of the decoration proteins binding to these A-loops should thus also be conserved in these phages. Alignment of the N-terminal domain sequences from the 17 corresponding decoration proteins (Fig. 3a) revealed that, whereas 25% of the first 24 residues are identical in all phages, only 8% of the last 40 residues are identical. This striking conservation difference between the region of helices α1 and α2 and the region of helices α3, α4 and α5 suggested that the first two helices play a critical role in capsid binding. Consistently, a quadruple mutation in helices α4 and α5 did not impact capsid binding. Moreover, α-helices located at the terminus of the decoration protein were also suggested to be responsible for capsid binding in phages T4^9^ and P4^6^.

In pb10 N-terminal helices, only two conserved residues are solvent-exposed in the NTD structure: the strictly conserved F22 and the partially conserved H19, substituted by a serine in two more distant phages (Fig. 3b). More extensive interaction of these helices with the capsid might be achieved through a conformational rearrangement of the α-helical domain, which would unveil conserved residues responsible for capsid recognition. Supporting this conformational change, we noticed that helix α5 that interacts with helices α1 and α2 in the NTD structure is in conformational exchange within full-length pb10. Such an exchange might favour conformational rearrangement upon binding. The fact that binding is associated to conformational changes might explain the low impact on capsid binding of mutations affecting pb10 solvent exposed residues.

Similar irreversible interactions were described for other phage structural proteins, like the head completion proteins that are integrated into the viral particle through a tightly regulated assembly pathway^31^. During assembly, large conformational rearrangements are observed, and the resulting assembled viral complexes (heads, tails) are extremely difficult to dissociate^32^. Such interaction, combining cooperativity and avidity processes, is for the first time measured and structurally characterized in the case of a decoration protein binding to a capsid. Our data suggest that irreversible binding of the pb10 decoration protein provides an advantage to survive in harsh environmental conditions that can be found in the diverse ecosystems colonized by the phages and their hosts. We surmise that binding of pb10 reinforces the capsid shell against rearrangements induced by modifications of pH or salt concentration at the intercapsomer interfaces or against the mechanical constraints induced by fluctuations in the packing of the highly condensed DNA molecule.

## Methods

### Construction of pb10 expression vectors and protein purification

The coding sequence of full-length pb10 as well as separate NTD and CTD (GenBank accession: AAU05286) were cloned into the pET28b vector in frame with a C-terminal His-Tag by using a common PCR and restriction strategy. The NTD includes the first 77 amino-acids, while the CTD includes residues 72 to 164. Site-directed mutagenesis (Quickchange Kit, Stratagene) was used to generate the expression vectors of the different mutants of pb10 investigated in this study. Expression and purification of the different versions of pb10 are detailed in SI.

### SAXS analysis of pb10

Small angle x-ray scattering (SAXS) experiments were carried out at the SOLEIL synchrotron SWING beamline (Saint-Aubin, France). The sample to detector (Aviex CCD) distance was set to 1790 mm, allowing reliable data collection over the momentum transfer range 0.008 Å^−1^ < q < 0.5 Å^−1^ with q = 4πsinθ/λ, where 2θ is the scattering angle and λ is the wavelength of the X-rays (λ = 1.0 Å). In order to separate potential aggregates from isolated proteins, SAXS data were collected on samples eluting from an online size-exclusion high-performance liquid chromatography column (SE- HPLCBio-SEC3, Agilent) available on SWING and directly connected to the SAXS measuring cell (more details in SI).

### Capsid and phage production

Phage T5 expanded capsids were prepared as previously described^14^ and dialyzed against Phosphate Buffer Saline pH 7.4 (PBS) for decoration assays. Their concentration was determined as described in SI. T5 DNA-filled capsids (heads) were produced by infection of E. coli strain F with T5D18amH5 defective in tail assembly^33^. Mutant phages T5Δ*dec* and T5*D18 am*-Δ*dec* producing phage or separate heads lacking pb10 were constructed as described in SI. Phage and heads were concentrated by precipitation in NaCl-PEG and then purified on cesium chloride gradient. Their concentration was determined as follows: [particles/mL] = 3.4 × 10^11^ × OD_260_.

### Binding assays by native agarose gels

Purified capsids were mixed with various amounts of pb10 at a final capsid concentration of 4 nM and incubated at 4 °C for 30 min. The samples were loaded on a 1.5% agarose gel in TAMg buffer (40 mM Tris-HCl, 20 mM acetic acid, 1 mM MgSO_4_, pH around 8.1) and migrated at 25 V overnight. The capsid bands were coloured with Coomassie blue. The molar concentration of binding sites was calculated by multiplying the capsid concentration by 120 sites per capsid.

### NMR experiments and data analysis

NMR experiments were performed at 20 °C on Bruker DRX600 and DRX700 spectrometers equipped with triple resonance cryoprobes. NMR data were processed with Topspin 1.3 software (Bruker Biospin, Germany) and analysed with CcpNmr Analysis software^34^. The backbone resonances of the separate pb10 domains were assigned using a standard procedure based on the analysis of 2D ^1^H-^15^N HSQC and 3D ^1^H-^15^N-^13^C CBCA(CO)NH, HNCACB, HNCO, HN(CA)CO, HBHA(CO)NH and HN(CA)NNH experiments. The backbone chemical shifts could be assigned for 66 residues out of 86 in the NTD and 88 out of 102 in the CTD. Side-chain resonances of separate domains were assigned using 2D homonuclear and 3D heteronuclear TOCSY and NOESY experiments. The side-chain chemical shifts could be assigned for 74 and 96 residues in the NTD and CTD, respectively.

### Structure calculation of separate pb10 domains

In-house INCA program^35^ was used for NOE assignment and structure calculation. Phi/psi angles were predicted using TALOS+^36^ and Preditor^37^ softwares. Backbone hydrogen bonds constraints were identified by recording a 2D ^15^N-^1^H HSQC experiment 1 h after solubilisation in D_2_O of the lyophilized ^15^N-labelled protein. The 20 lowest energy structures were selected as the NMR structure ensemble.

### Pb10 structure and dynamics analysis

The 3D structure of the full form of pb10 was determined using a simulated annealing protocol with the XPLOR-NIH 2.36 software1.2^38^. NOE-derived inter-proton distances, chemical shift-derived dihedral angles and small angle X-ray diffusion (SAXS) data were used as restraints during this protocol. Distance and dihedral angle restraints were those obtained for the separate domains with the exception of the restraints involving residues with altered ^15^N or ^1^H chemical shifts in pb10 as compared to the separate domains (Supplementary Fig. S3). Thus 150 structures were generated and the 30 best structures were kept for refinement. In both annealing and refinement protocols, SAXS data were taken into account via a specific potential term. The final structures showed no distance restraint violation larger than 0.5 Å and no dihedral angle restraint violation larger than 10 °. To score the consistency with the SAXS data, a chi value was calculated as the square root of 1/N * Σq((Iexp-Icalc)/σ)^2^, Iexp being the experimental intensities, Icalc the intensities calculated from the model using Crysol, σ the experimental error and N the number of points. The 20 final models were selected as those with the best chi values. The average chi value was 2.7 ± 0.1 for the 20 models. More details are given in Supplemental Information.

Standard ^1^H ^15^N NMR experiments^39^ were used to determine ^15^N R_1_, ^15^N R_2_ and {^1^H-^15^N} NOE parameters. These experiments were analysed using CcpNmr Analysis^34^. ^15^N R_1_ and ^15^N R_2_ experiments were performed in duplicate on the full-length protein only. {^1^H-^15^N} NOE experiments were performed in duplicate on the separate domains and quadruplicate on the full-length protein. In order to interpret the measured relaxation parameters in terms of motions, S^2^, τe and Rex values were calculated using the Modelfree software^20^. Error bars on these values were given by Monte-Carlo simulations based on ^15^N R_1_, ^15^N R_2_, and {^1^H-^15^N} NOE parameters comprised within their own error bars.

### Cryo-electron microscopy and image reconstruction

3 μl of sample was deposited on Quantifoil R2/1 copper grids (Quantifoil Micro Tools GmbH) that had been glow-discharged for 10 seconds. Grids were blotted and plunge-frozen into a liquid nitrogen-cooled mix of 2:1 ethane and propane using an FEI Vitrobot Mk III (FEI, Hillsboro OR). Grids were mounted in an FEI Krios microscope (expanded capsid without pb10) or an FEI Polara microscope (expanded capsid with pb10), both operated at 300 kV and imaged at nominal magnifications of 75,000× or 78,000× for the Krios and Polara respectively. Images were collected using the FEI “EPU” automation software on an FEI Falcon 2 direct electron-detecting camera with 2 second exposures. For the expanded capsid without pb10, 5,171 images were taken and 26,027 particles were selected for the final icosahedral reconstruction done with Auto3dem^40^. For the expanded capsid with pb10, 1939 images were taken and 1,856 particles were selected for the final icosahedral reconstruction. The CTF was determined with the ctffind3 software^41^ and the final resolutions, estimated by Fourier shell correlation (FSC = 0.5), are 9 Å and 8 Å for the capsids with and without pb10, respectively. UCSF Chimera^42^ was used to visualize the density maps and to rigidly fit the pb8 model developed using the phyre2 server (Huet *et al*.^10^). VMD^43^ and the MDFF plugin^44^ were used to improve the model through flexible fitting and the APBS plugin^45^ was used to determine the electrostatic properties (with a mobile ion concentration of 150 mM) of the pb8 and the pb10 models.

### Surface Plasmon Resonance Binding Assays

SPR experiments were performed on a T200 instrument (GE Healthcare) equilibrated at 25 °C in PBS supplemented with 1 mg/mL BSA (to prevent unspecific binding events). The surface of a CM5 sensor chip was functionalized with rabbit antibodies raised against empty T5 capsids using a standard amine coupling procedure, resulting in a density of around 10,000 resonance units (RU) of covalently immobilized IgGs. The anti-capsid sensor chip was used to capture T5 capsids thus leading to the regeneratable binding surface used to study the interactions with pb10. Purified T5 capsids (0.2 mg/mL) were injected at 5 μL/min yielding a capsid density of 800-1, 200 RU. The sensor surface could be regenerated with a solution of 0.85% phosphoric acid without loss of T5 capsid binding capacity. Decoration protein binding assays were carried out using solutions of pb10 at concentrations ranging from 0.09 to 40 nM, that were injected for 200–360 s at 50 μL/min. Dissociation was then monitored for 600 s by flowing running buffer at 50 μL/min.

The real-time SPR sensorgrams were double-referenced, i.e. both the pb10 responses on the anti-capsid surface (with no T5 capsid captured) and the buffer response on the captured capsids were subtracted. For optimal confidence, the dissociation rate k_off_ of the pb10/capsid complexes was determined first, by deconvoluting it from the release of capsid from the anti-capsid surface. The average k_off_ value (2 × 10^−4^ s^−1^), similar for all pb10 variants, was then set as a constant, and both the association rate constants k_on_ and the equilibrium dissociation constant K_D_ were determined by globally fitting the curves corresponding to each experiment to a simple Langmuir mass transport-limited model using the BIAevaluation 4.1 software (GE Healthcare). Mass transport limitation was demonstrated by the flow-dependence of association profiles (flow range from 10 to 100 μL/min).

### Phage thermostability assays

For fluorescent assays, T5 wt or T5Δdec phages were diluted to a final concentration of 5 × 10^9^ pfu/mL in buffer solutions containing SYBR-Gold (diluted 3,000X from a 10,000X stock solution of SYBR^®^ Gold in DMSO, Invitrogen). SYBR-Gold exhibits a 1,000-fold fluorescence enhancement upon binding to nucleic acids, which allows to quantify free DNA in solution. Reaction mixtures were made in quadruplicate in a 384-well fast PCR plate at a final volume of 10 μL. Temperature was increased from 4 °C to 99 °C at 3 °C/min with a QuantStudio 12 K Flex instrument (LifeTechnologies). Fluorescence was recorded as a function of temperature in real time (excitation with a white LED source and emission filtered through a Vic emission filter). The DNA exit temperature (T_ex_), assessed by the release of DNA, was calculated with QuantStudio 12 K Flex software v1.2.2 as the maximum of the derivative of the resulting SYBR-Gold fluorescence curves. For EM imaging of DNA-free phages, particles heated at 1 °C/min up to 78 °C were cooled at room temperature and stained with 1% uranyl acetate as described in SI.

## Additional Information

**Accession Codes**: The NMR structures of the NTD and CTD were deposited in the Protein Data Bank (codes 5LXL and 5LXK, respectively). The NMR data used to build these structures were deposited in the Biological Magnetic Resonance Data Bank (entries 34047 and 34046, respectively). The cryo-EM density maps were deposited in the EMDB with the accession codes EMD-8419 and EMD-8423 for the model with and without pb10 respectively. The structural model of the hexamer was deposited in the PDB with the accession code 5TJT.

**How to cite this article**: Vernhes, E. *et al*. High affinity anchoring of the decoration protein pb10 onto the bacteriophage T5 capsid. *Sci. Rep.* **7**, 41662; doi: 10.1038/srep41662 (2017).

**Publisher's note:** Springer Nature remains neutral with regard to jurisdictional claims in published maps and institutional affiliations.

## Supplementary Material

Supplementary Information

## Figures and Tables

**Table 1:** Affinity constants of pb10 variants for T5 empty expanded capsids determined by SPR.

Protein	k_on_ (10^6^ M^−1 ^s^−1^)	k_off_ (10^−4^ s^−1^)^a^	K_D_ (pM)^b^	Reduced affinity	Correctly folded
pb10	180 ± 64.8	2	1.11	no	**yes**
pb10 (300 mM NaCl)	111	2	1.80	no	
pb10 (500 mM NaCl)	47.6	2	4.20	**yes**	**yes**^c^
pb10 (750 mM NaCl)	9.01	2	22.2	**yes**	
NTD	362 ± 122	2	0.55	no	
F36A	866 ± 122	2	0.23	no	**yes**
R38A	120 ± 14.6	2	1.67	no	**yes**
R38E	84.7 ± 10.3	2	2.36	no	**yes**
Y34A-R38E	0.417	2	480	**yes**	no
F36A-R38A	61.1 ± 13.2	2	3.27	**yes**	no
F36A-R38E	19.5 ± 3.80	2	10.3	**yes**	no
F11A, F22A				**yes**	no
K14A, K29A, K51A, K55A, K62A				no	
Y34A, R39A, R50E, K54E				no	**yes**
R38A-R39A, R50A-K51A, K54A-K55A				no	
R38A-R39A-R50A-K51A				no	**yes**^c^

^a^The k_off_ was set at 2 × 10^−4^ s^−1^ for all proteins (see Methods).

^b^No errors are indicated for the K_D_ because the k_off_ was forced at 2 × 10^−4^ s^−1^.

^c^Structure verified by SAXS.

All experiments were performed at least in duplicate except for wild-type pb10 at high NaCl concentrations and Y34A-R38E mutant due to lack of material. For fifteen of the pb10 mutants (last 5 lines) the affinity for T5 capsid was compared to that of wild-type pb10 on agarose gels (see Supplementary Fig. S8) and not measured by SPR.

**Table 2:** Temperature of DNA exit (T_ex_) assessed by fluorescence thermostability assays.

	MgCl_2_	NaCl (mM)	Hepes pH 8.2	Tris pH 7.2	Hepes pH 7.0	Cacodylate pH 6.0	Acetate pH 5.0
T5 wt	1 mM	0	68.7	73.5	72.6	74.2	71.0 ± 1.3
		100	66.9	71.5	70.0	71.9	63.9
		300	66.5	66.0	67.3	65.4	59.1
T5*∆dec*	1 mM	0	69.3	73.6	73.1 ± 0.7	73.7	70.3 ± 0.9
		100	67.3	71.5	70.0	71.9	62.2
		300	64.8	64.2	65.3	62.8	54.3
		***∆T***_***ex***_ (***300***)	**1**.**7**	**1**.**8**	**2**.**0**	**2**.**6**	**4**.**7**
T5 wt	0.1 mM	0	68.2	72.7	72.2	72.7	70.2 ± 2.0
		100	66.5	70.8	69.8	71.4	60.4
		300	60.3	60.3	60.7	59.8	53.8
T5*∆dec*	1 mM	0	68.9	72.4	72.5	72.6	67.2 ± 0.7
		100	66.7	71.2	69.6	70.9	58.0 ± 0.4
		300	56.1	57.2	57.8	56.2	47.5
		***∆T***_***ex***_ (***300***)	**4**.**2**	**3**.**1**	**2**.**9**	**3**.**6**	**6**.**3**

T5 wt or T5Δ*dec* were heated in different buffer solutions at 3 °C/min in the presence of SYBR-Gold (see Fig. 8a) and the temperature of DNA exit *T*_*ex*_ was determined as described in Experimental Procedures. The difference in *T*_*ex*_ between T5∆*dec* and T5wt is indicated for buffers containing 300 mM NaCl. Except otherwise indicated, the standard deviation on *T*_*ex*_ value was less than 0.3 °C.
