# Erratum: High affinity anchoring of the decoration protein pb10 onto the bacteriophage T5 capsid

**DOI:** 10.1038/srep43977

**Published:** 2017-04-28

**Authors:** Emeline Vernhes, Madalena Renouard, Bernard Gilquin, Philippe Cuniasse, Dominique Durand, Patrick England, Sylviane Hoos, Alexis Huet, James F. Conway, Anatoly Glukhov, Vladimir Ksenzenko, Eric Jacquet, Naïma Nhiri, Sophie Zinn-Justin, Pascale Boulanger

Scientific Reports 7: Article number: 4166210.1038/srep41662; published online: 02 06 2017; updated: 04 28 2017

This Article contains typographical errors.

In the results section under subheading **‘The C-terminal domain adopts an immunoglobulin fold in solution’**,

“A search for structurally similar proteins possessing using the Dali server identified many proteins containing an Ig-like fold, including immunoglobulin receptors, fibroblast growth factor receptors and the T-cell surface glycoprotein CD4.”

should read:

“A search for structurally similar proteins using the Dali server identified many proteins containing an Ig-like fold, including immunoglobulin receptors, fibroblast growth factor receptors and the T-cell surface glycoprotein CD4.”

In Table 2 in the MgC_l2_ column, the second T5*∆dec* value ‘1mM’ should read ‘0.1mM’.

