# Supplementary material for: High affinity anchoring of the decoration protein pb10 onto the bacteriophage T5 capsid
Source: Sci Rep. 2017 Feb 6;7:41662. doi: 10.1038/srep41662 (PMC5292684; doi:10.1038/srep41662)
Supplement: Supplementary Information [file srep41662-s1.pdf]

SUPPLEMENTARY INFORMATION

High affinity anchoring of the decoration protein pb10 onto the bacteriophage T5 capsid

Emeline Vernhes, Madalena Renouard, Bernard Gilquin, Philippe Cuniasse, Dominique Durand, Patrick England, Sylviane Hoos, Alexis Huet, James F. Conway, Anatoly Glukhov, Vladimir Ksenzenko, Eric Jacquet, Naïma Nhiri, Sophie Zinn-Justin* and Pascale Boulanger*

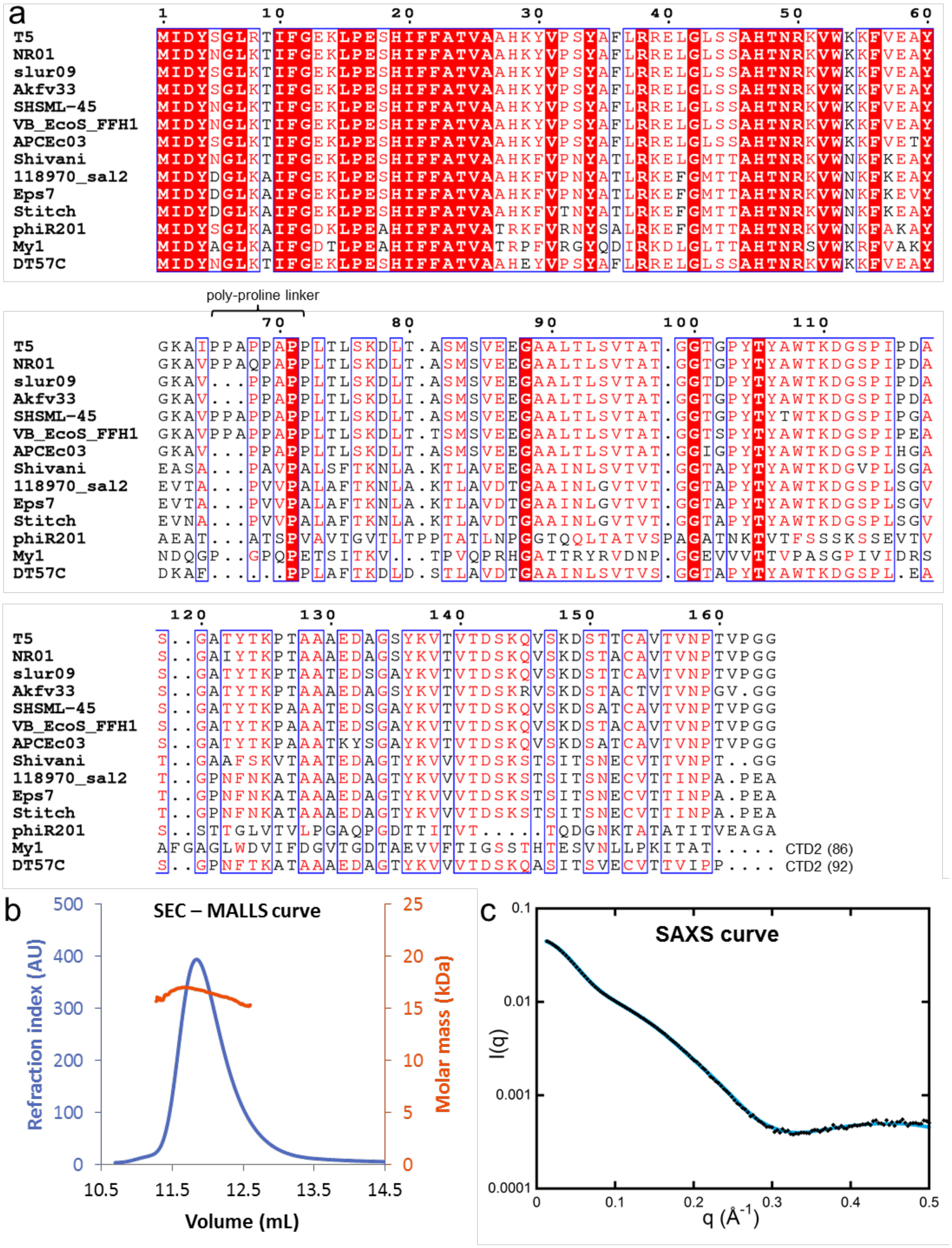

a
T5
NR01
slur09
Akfv33
SHSML-45
VB_EcoS_FFH1
APCEc03
Shivani
118970_sal2
Eps7
Stitch
phiR201
My1
DT57C
poly-proline linker
CTD2 (86)
CTD2 (92)
b
SEC – MALLS curve
Refraction index (AU)
Molar mass (kDa)
Volume (mL)
c
SAXS curve
I(q)
q (Å-1)

Supplementary Figure S1: The decoration protein pb10 is an elongated monomer composed of two domains separated by a poly-proline linker.

(a) Multiple sequence alignment of the two-domain decoration proteins of T5-like bacteriophages infecting the enterobacteria *E. coli* (T5, Akfv33, APCEc03, Shivani, vB_EcoS_FFH1, EPS7, DT57C), *Salmonella* (NR01, Slur09, Stitch, 118970_sal2), *Yersinia* (phiR201), *Shigella* (SHSML-45) and *Pectobacterium* (My1), as calculated using MAFFT through the Consurf webserver[1]. The alignment figure was generated using ESPript 3.0[2]. Residues strictly conserved in all phage protein sequences are displayed on a red background. Residues conserved at more than 70% are displayed in red on a white background. Non-conserved residues are displayed in black. Decoration proteins from My1 and DT57C possess a third domain indicated as CTD2 (number of residues in brackets). The CTD2 from DT57C is 95 % identical to the CTD of T5. The CTD2 from My1 is more divergent and could not be aligned with T5 CTD.

(b) SEC-MALLS analysis of pb10. Pb10 elutes as a monomer in a single peak (blue) corresponding to about 17 kDa (as indicated by the red curve).

(c) Comparison between the experimental (black dots) and the calculated (blue line) SAXS curves. The calculated curve was obtained using Crysol[3] based on the GASBOR[4] model shown in Fig. 1B.

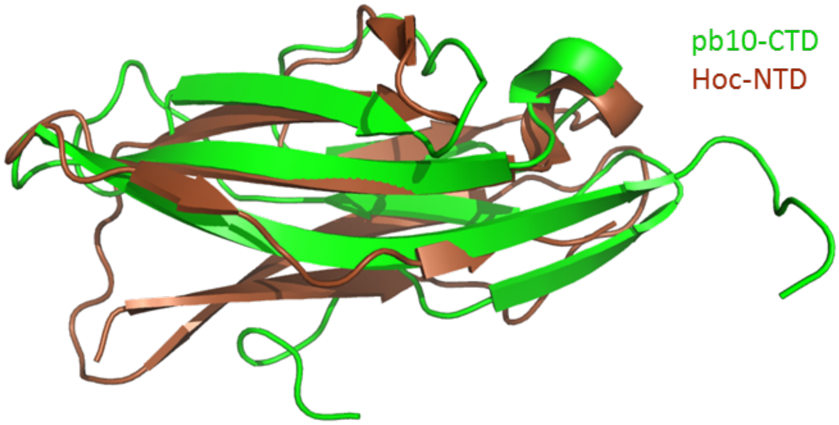

Supplementary Figure S2: Superimposition of the 3D structure of pb10 CTD (green) with that of the N-terminal domain of the Hoc protein from phage RB49 (brown; PDB entry: 3HSH).

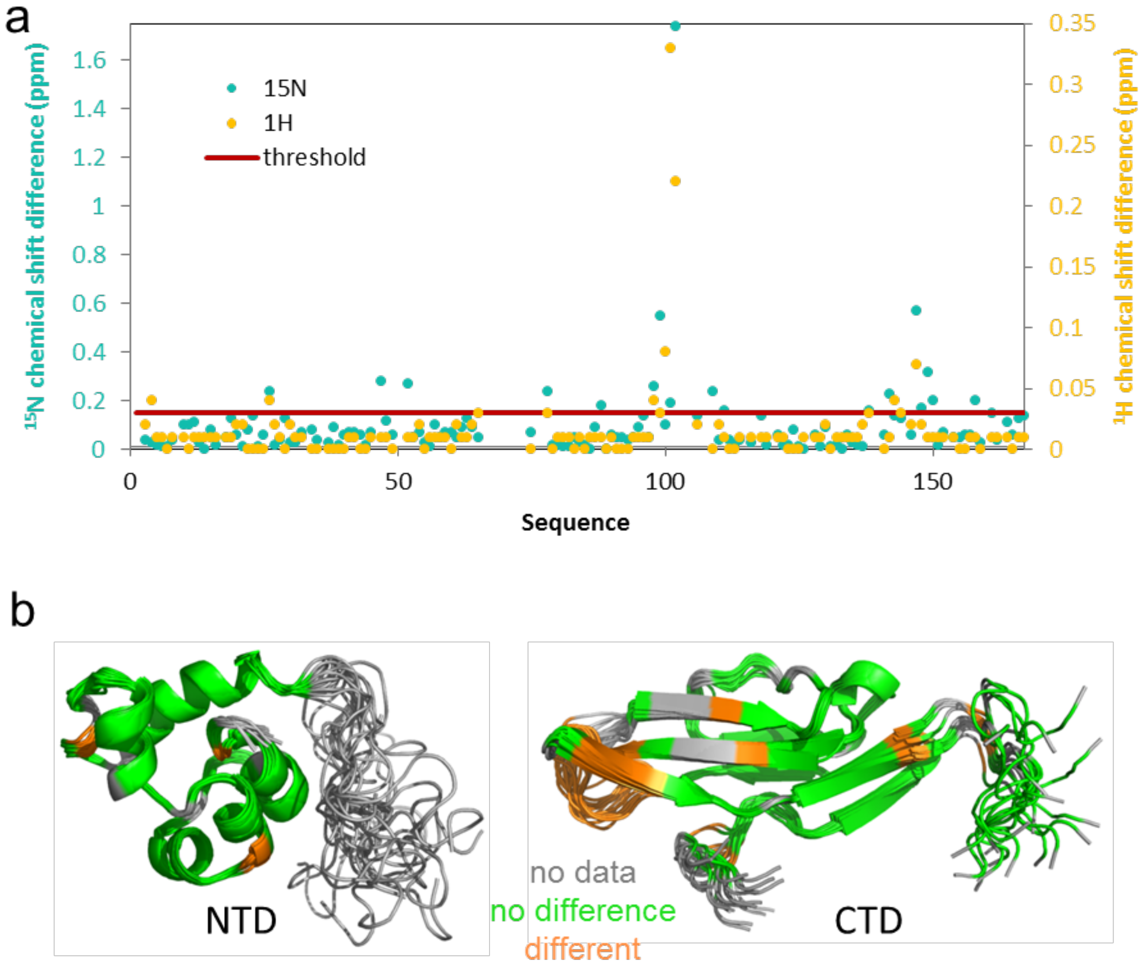

Supplementary Figure S3: Structure conservation of the isolated domains compared to full-length pb10.
(a) Chemical shift differences between full-length pb10 and its isolated domains. The threshold was calculated based on the resolution of the NMR spectra.
(b) Structures of the isolated NTD and CTD with residues coloured according to the chemical shift differences between full-length pb10 and its isolated domains: absence of data in grey, no significant difference (lower than the threshold) in green, a significant difference (higher than the threshold) in orange.

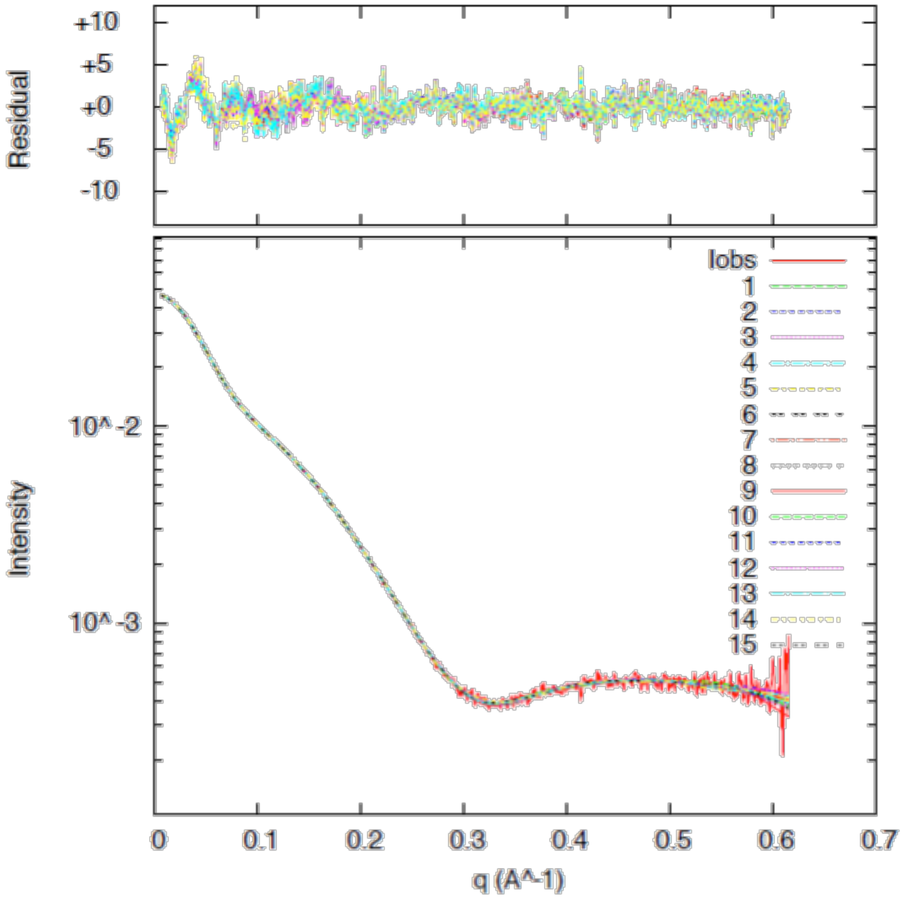

Supplementary Figure S4: Comparison between the experimental (red line) and calculated SAXS curves. The calculated curves correspond to the 15 best structures of pb10 determined using a subset of the NMR NOE and dihedral angle restraints obtained for the isolated NTD and CTD together with SAXS data obtained on the full length pb10. In the upper panel, the residual is calculated according to $R(q)= (I_{calc(}q) – I_{obs}(q))/I_{err}(q)$ where I_{calc}, I_{obs} and I_{err} correspond to the calculated SAXS intensity, the experimental intensity and the associated error, respectively.

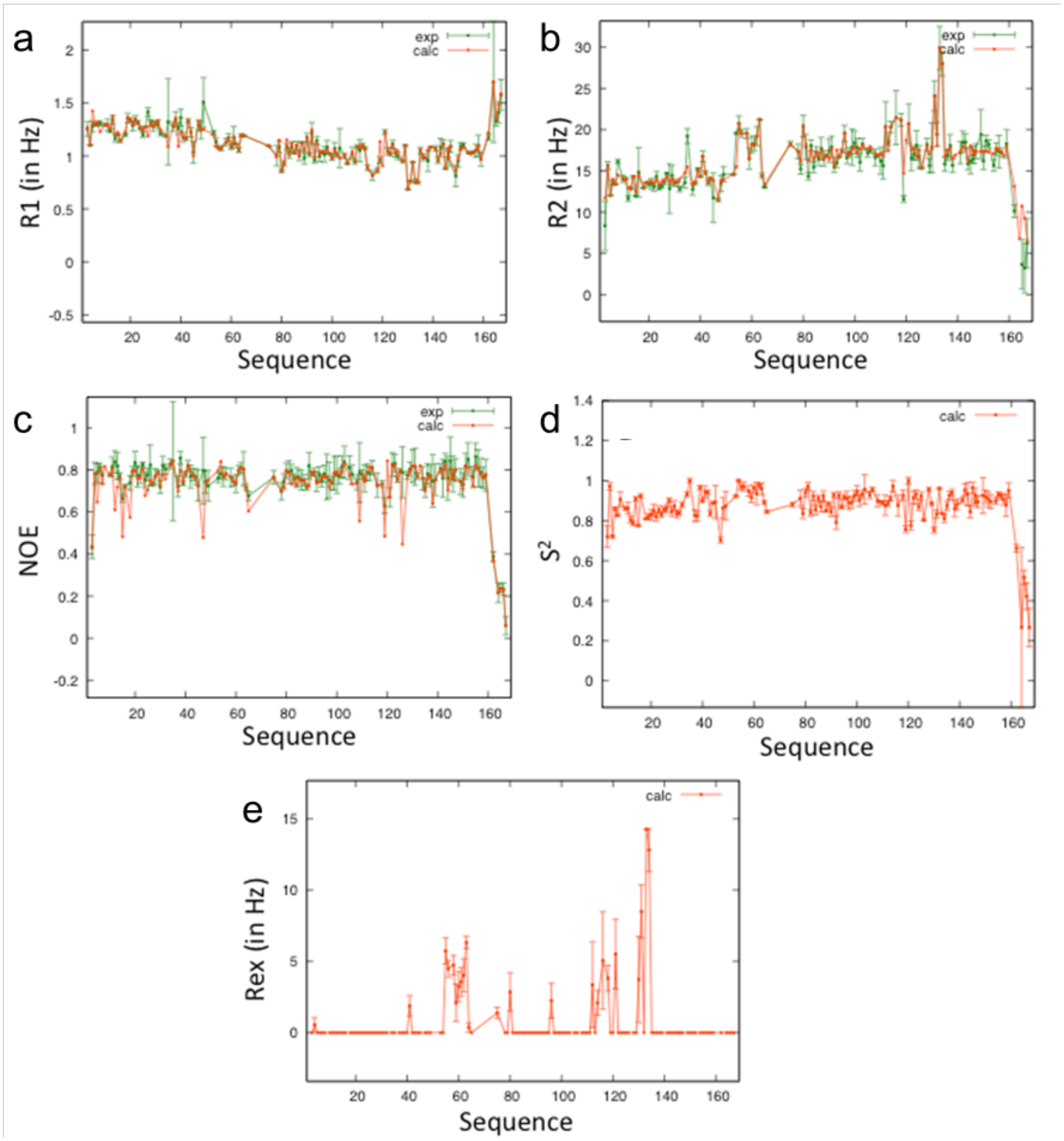

Supplementary Figure S5: Modelfree analysis of the pb10 ^{15}N relaxation parameters in terms of physical motions[5]. The experimental ^{15}N **(a)** R_1, **(b)** R_2 and **(c)** ^{1}H->^{15}N NOE values measured at 700 MHz were fitted using a motion model including global correlation times $\tau_{c(NTD)}$ (10.0 ns) and $\tau_{c(CTD)}$ (12.2 ns) for each domain determined from the R2/R1 ratio, **(d)** a local motion order parameter S^2, a local motion correlation time τ_e and **(e)** a slow exchange contribution to R_2 named R_{ex}. The experimental and calculated values are displayed in green and orange, respectively.

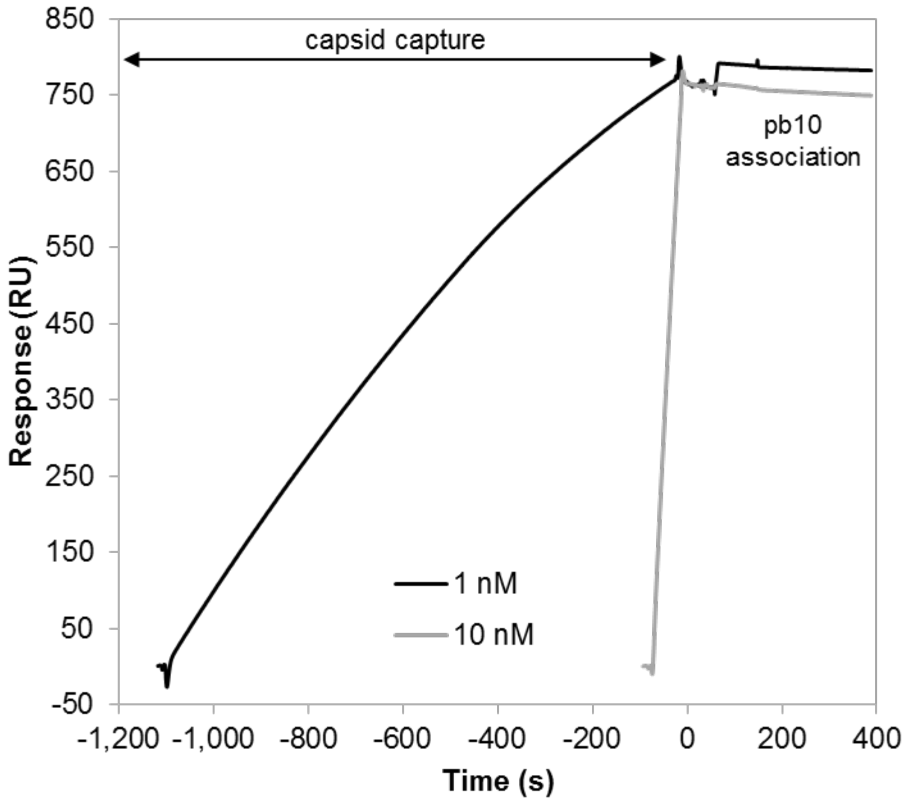

Supplementary Figure S6: Detection of partially decorated capsids.
Expanded capsids (32 µg/ml), incubated with limited amounts of pb10-His6 (1 and 10 nM, respectively $6.8x10^{-3}$ and $68x10^{-3}$ pb10/hexamer), were captured on the surface of a nickel activated NTA (Ni^{2+}-NTA) sensorchip. They were then saturated with untagged pb10 (100 nM). Many available pb10 binding sites are observed in the case where capsids were incubated with pb10-His6 1nM (response of 35 RU for 750 RU of capsid), while very few pb10 sites remain available when capsids were incubated with pb10-His6 10nM (4 RU for 750 RU of captured capsid).

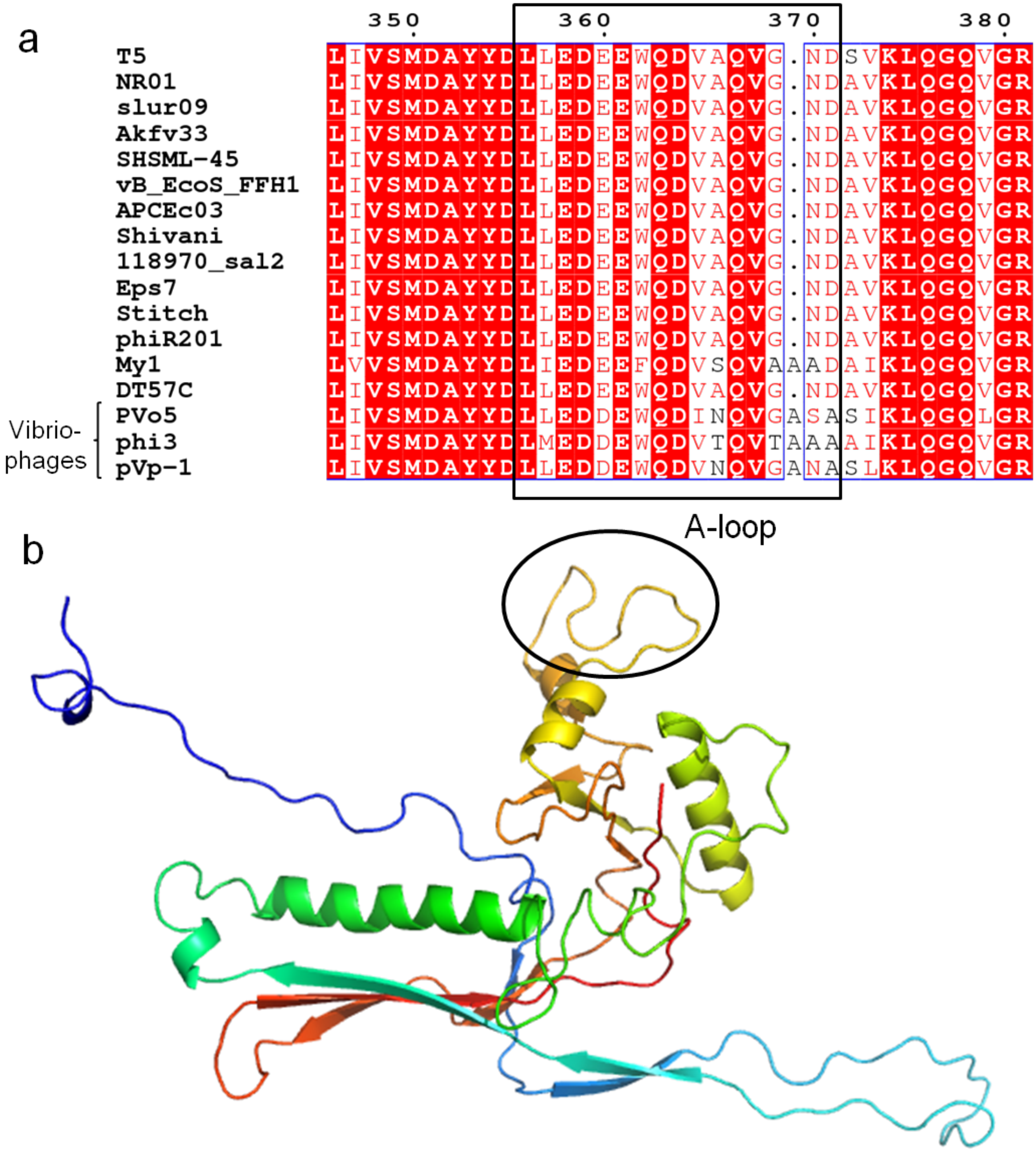

Supplementary Figure S7: (a) Sequence alignment of the major head proteins from 17 T5-like (closely-related and more distant) phages. The A-loop region is highlighted in the black box. The alignment figure was generated using ESPript 3.0[2].
(b) Structural model of the major head protein pb8 of phage T5 coloured from blue (N-terminus) to red (C-terminus). The A-loop is located within the black oval.

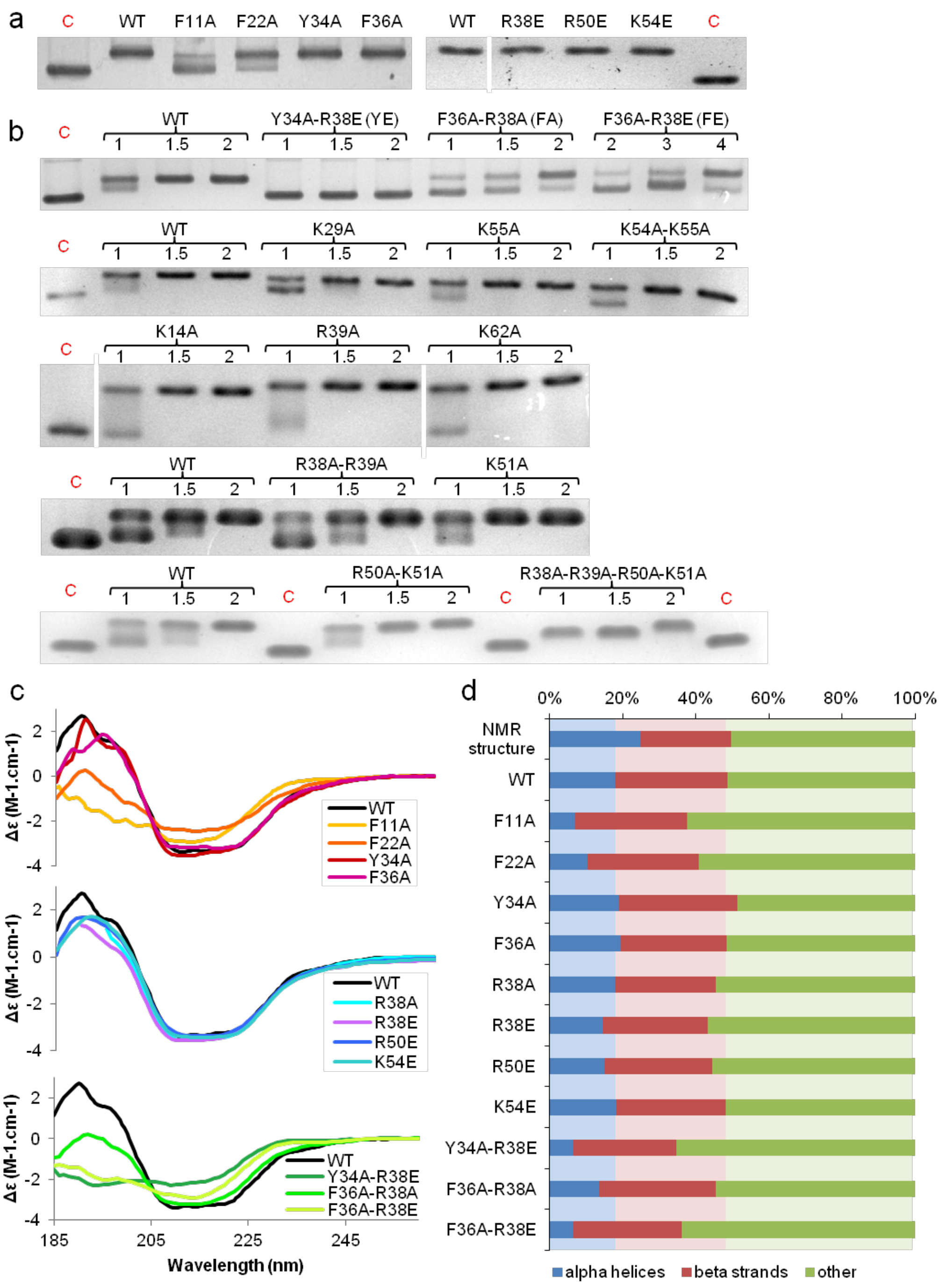

Supplementary Figure S8: The affinity of pb10 for capsids is reduced only when the secondary structure of the binding domain is altered.

(a, b) Native agarose gels of expanded capsids alone (indicated with a red "C") or pre-incubated with wild-type pb10 (WT) or pb10 mutants are displayed either **(a)** at a [pb10]/[hexamer] molar ratio of 2 or **(b)** with increasing [pb10]/[hexamer] molar ratios.

(c) CD spectra of wild-type pb10 in black and pb10 mutants in other colours as indicated. The spectra are presented on 3 separated panels for clarity.

(d) Secondary structure contents of wild-type pb10 and pb10 mutants as predicted by the SELCON3, CONTIN and CDSSTR algorithms available on the DichroWeb server[6] and the BeStSel algorithm[7].

Supplementary Table S1: Structural statistics corresponding to the 3D structures of pb10 NTD and CTD.

**Domain**	**NTD**	**CTD**
**Distance restraints**		
NOE		
Assigned peaks	2,423	2,168
Intra-residual restraints	579	580
Sequential restraints	170	336
Medium range restraints (2-4)	126	110
Long range restraints (>5)	138	307
Ambiguous restraints	290	163
Hydrogen bonds restraints	30	60
Dihedral restraints	118	152
**Violations**		
number of NOE restraints > 0.5	0 ± 0	0.1 ± 0.3
number of dihedral restraints > 10°	0 ± 0	0 ± 0
**Rms-deviation from ideal geometry**		
bond lengths (Å)	0.0188 ± 0.0002	0.018 ± 0.0002
bond angles (°)	3.68 ± 0.05	3.68 ± 0.03
improper angles (°)	2.40 ± 0.16	3.54 ± 0.12
**Energy (kcal/mol)**		
E overall	1,227 ± 22	1,256 ± 18
E bonds	133 ± 2	136 ± 3
E angles	503 ± 12	516 ± 9
E impropers	13.3 ± 0.6	25.3 ± 1.1
E_van der Waals	185 ± 13	202 ± 13
Electrostatic[a]	-177 ± 17	-307 ± 14
**Ramachandran plot statistics (%)[b]**		
Residue in most favoured regions	89	79.7
Residue in additional allowed regions	10.9	15.6
Residue in generously allowed regions	0.1	1.9
Residue in disallowed regions	0	2.8
Rms diff. to the mean structure (Å)[c]	0.43 ± 0.08	0.44 ± 0.08

[a] Electrostatic energy was calculated with CHARMM[8] using Charmm22 parameters and a distant dependent dielectric constant.

[b] The percentage corresponds to residues 4-68 and 79-160 for N77 and C72 respectively. They were determined by PROCHECK[9,10].

[c] The rms values correspond to residues 4-68 and 79-160 for N77 and C72 respectively. They were calculated on the backbone atoms C, N and Cα.

Supplementary Table S2: Structural statistics for the ensemble of 15 selected conformers of pb10.

**Number of restraints:**	
Distance restraints	2,490
Dihedral angle restraints	214
Hydrogen bonds restraints	70
**NOE distance violations (> 0.5Å)**	20.9 ± 6.5
**Hydrogen bond distance restraints**	11.8 ± 2
**RMSD from idealized covalent geometry**	
Bonds(Å)	0.009
Angles (°)	1.076
Improper (°)	0.852
RMSD on restraints	
Distance (Å)	0.118
Dihedral (°)	3.81
Hydrogen bond distance (Å)	0.338
Coordinates Precision (Å):	
Residue (10-64)	1.39 ± 0.32
Residue (80-96+106-140+151-158)	1.08 ± 0.51
Ramachandran plot (residues 10-65;79-168)[a]:	
Most favoured (%)	83.0
Additional allowed regions (%)	13.0
Generously allowed regions (%)	2.0
Disallowed regions (%)	2.0

[a] After relaxation in the Charmm22 force field

Supplementary Methods

Protein production and purification. *E coli* BL21(DE3) cells harbouring each of the different pb10 expression vectors were grown at 37°C in 2-YT medium supplemented with 50 µg/mL kanamycin. For NMR studies, ^{15}N or $^{15}N/^{13}C$-labeled proteins were produced by growing *E coli* BL21(DE3) cells in M9 minimum medium containing $(^{15}NH_4)_2SO_4$ as the only nitrogen source and/or ^{13}C-glucose as the only carbon source (Cambridge Isotope Laboratories, Inc.). At mid-exponential growth phase (OD_{600}=0.6-0.8), protein expression was induced by addition of 0.4 mM isopropyl-β-D-thiogalactopyranoside (IPTG) and the growth continued for 3h at 37 °C. Bacterial cells harvested by centrifugation were suspended in 20 mL of lysis buffer (50mM Tris-HCl pH 7.2, 150 mM NaCl, 1 mM EDTA, 0.6% Nonidet P-40, 0.1 g/L lysozyme) and incubated at 37°C for 10 min. DNAse (Universal Nuclease, Pierce) and 1 mM $MgCl_2$ were then added for a 10 min additional incubation and the suspension was centrifuged at 20,000g for 30min at 4°C. The supernatant was loaded onto a 5 mL HisTrap™ FF column (GE Healthcare) pre-equilibrated in either 25 mM Tris-HCl pH 8.0 or 50 mM Hepes pH 7.0 containing 150 mM NaCl that was connected to an ÄKTA purifying system. The column was extensively washed with the loading buffer supplemented with 1M NaCl and pb10 or derivative proteins were eluted with a 0-1 M imidazole gradient. The eluted fractions were collected and proteins further purified by cation or anion exchange chromatography, depending on their calculated isoelectric point. Full-length pb10 (pI=7.89) and pb10-NTD (pI=9.75) were loaded onto a 5 mL HiTrap SP column (GE Healthcare) pre-equilibrated in 50 mM Hepes buffer pH 7.0. Pb10-CTD (pI=5.76) mL was loaded onto a 5 mL HiTrap Q HP column (GE Healthcare) pre-equilibrated in 25 mM Tris-HCl pH 8.0. The proteins were eluted with a 0-1 M NaCl gradient. Purified proteins were dialyzed against 25 mM sodium phosphate pH 7.2 and concentrated if needed on a centrifugal filter (Amicon Ultra-4, 10 kD, Millipore) to a final concentration of at least 200 µM.
Untagged pb10 was produced from a pET28 expression vector including a sequence coding the TEV protease recognition site, ENLYFQS, upstream of the C-terminal His-tag of pb10. After a first purification step on an HisTrap™ FF column as described above, the pb10-TEV-His protein was dialyzed in 25 mM Tris Buffer pH 8.0 containing 0.5 mM EDTA, 1mM DTT and 150 mM NaCl. The His-tag was then cleaved by incubating the protein with purified His-tagged TEV protease (prepared in our laboratory) at 4°C overnight with a ratio of 1 OD_{280nm} of TEV protease for 10 OD_{280nm} pb10-TEV-His protein. Purification of cleaved pb10 and removal of His-TEV protease was achieved in one step by loading the reaction mixture onto a 5 mLHisTrap™ FF column and harvesting of untagged pb10 in the flow-through.

SEC-MALLS (Size-exclusion-chromatography-coupled with multi-angle laser light scattering). Chromatographic analysis of pb10 was carried out by SEC-MALLS using a GPCMax-TDA system (Viscotek, Malvern, France). A pb10 sample (200 µL at ≈ 4 mg/mL) was injected at a flow rate of 0.5 ml/min onto a Superdex 75 HR10/30 column (GE Healthcare) equilibrated with 25 mM sodium phosphate pH 7.2 containing 150 mM NaCl. Elution was monitored on-line by a UV-visible spectrophotometer, a differential refractometer, a 7° low angle light scattering detector, a 90° right angle light scattering detector and a differential pressure viscometer. The instrument was calibrated using bovine serum (Sigma–Aldrich). The OmniSEC program (Malvern) was used for the acquisition and analysis of the data.

Details of SAXS data processing. Purified pb10 protein was injected in a Superdex 75 column (GE Healthcare) that was pre-equilibrated with preparative buffer comprising 25 mM sodium phosphate buffer pH 7.0. Flow rate was 0.2ml/min, frame duration was 1.0 s and the dead time between frames was 0.5 s. For each frame, the protein concentration (about 3.5 mg/ml at the top of elution peak) was estimated from UV absorption at 280 and 295 nm using a spectrometer located immediately upstream of the SAXS measuring cell. Selected identical frames corresponding to the elution peak were averaged. A large number of frames were collected before the void volume and averaged to account for buffer scattering. SAXS data were normalized to the intensity of the incident beam and background (i.e. the elution buffer) subtracted using the programs FoxTrot (courtesy of SWING beamline) and Primus[11]. The scattered intensities were displayed on an absolute scale using the scattering by water.
The conformation of pb10 in solution was determined *ab initio* from the scattering curve using the program GASBOR that generates a volume filled with a compact chain of dummy residues. Models obtained from a hundred calculations are superimposed and compared using the program suite DAMAVER[12]. Their similarity is quantified using a normalized spatial discrepancy (NSD). The value found for NSD (0.96) indicates a high similarity between them.

Pb10 structure and dynamics analysis. The 3D structure of the full-length pb10 was determined using a simulated annealing protocol with the XPLOR-NIH 2.36 software1.2. All calculations were achieved on a cluster equipped with bi-pro Intel(R) XEON Nehalem X86 processors (I2BC, GIPSI, CEA). NOE-derived inter-proton distances, chemical shift-derived dihedral angles and small angle X-ray diffusion (SAXS) data were used as restraints during the simulated annealing protocol. A "synthetic" NOE distance set was built from those obtained for structure determination of the isolated N and C-terminal domains. The NOE-derived distances composing this "synthetic" set were the NOE distance restraints obtained for the isolated domains with the exception of those involving residues with altered ^{15}N or ^{1}Hn chemical shifts in pb10 as compared

to the isolated domains. This criterion was also applied to select a subset of φ, ψ dihedral restraints determined for the isolated domains using the TALOS software3. This led to a set of 2,490 NOE distance restraints and 214 dihedral angle restraints.

The structure determination was achieved in two steps. A high-temperature simulated annealing stage was run to obtain a good starting structure that was further used in the so-called refinement step to generate a large number of structures compatible with the NMR and SAXS data. The annealing step started with randomization of the torsion angles of an initial extended conformation of the protein. This structure was submitted to 1,000 steps of high-temperature (3,500 K) molecular dynamics. Temperature was controlled using the coupling to an external bath. During the high temperature molecular dynamics steps, radii were significantly reduced for all atoms except the CA and the experimental NOE distance restraints were taken into account via a soft potential and a scale factor of 2. The chemical shift derived-dihedral angle restraints were taken into account via a harmonic term with a scale factor of 10. Then the system was cooled to 25 K in steps of 12.5 K. At each temperature, 100 molecular dynamics steps were calculated. During cooling, the scale factor of the NOE restraints was gradually increased from 2 to 30 and the scale factor of the torsion angle was set to 200. The van der Waals radii of all atoms were increased during cooling via the rcon parameter that was decreased from its high temperature value 0.004 to 4 at the end of the cooling step. The atom-atom repulsive strength was also gradually decreased through the repel parameter from 0.9 to 0.8. During annealing, 150 structures were generated and the 30 best structures were kept for refinement. Refinement of the selected pb10 structures was achieved using a simulated annealing protocol essentially similar to the one used for the annealing step excepted that the starting structure was not a random one but the starting coordinates were those of the structures obtained via the annealing protocol.

In both annealing and refinement protocols, SAXS data were taken into account via a specific potential term. This term allowed minimizing the root mean square difference between the experimental intensities of the experimental SAXS spectrum and the intensities calculated from the Cartesian coordinates of a 3D structure of the protein. During all calculations, the X-ray scale factor was set to 400. 50 points were used for the calculation of each spectrum. The calculated intensities were corrected for higher accuracy by fitting of the experimental spectrum every 10th temperature. This included the evaluation of the solvent contribution.

Circular dichroism. The protein samples at 0.5 mg/mL were analysed on a Jasco J-180 spectropolarimeter at 20°C in 20 mM phosphate buffer at pH 7.0. Scans were performed between 185 and 260 nm with intervals of 1 nm at 50 nm/min. 3 to 8 accumulations were recorded for each protein. After buffer subtraction and conversion to international units (Δε) the spectra were smoothed to reduce noise.

Construction of T5Δ*dec* and T5*D18*am-Δ*dec* mutants. A deleted version of the *dec* gene encoding pb10 was constructed by PCR-based oligonucleotide-directed mutagenesis. The deletion encompassed 444 bp corresponding to codons 2-149 of the *dec* gene and the final PCR product included 484 bp and 558 bp flanking the 5'- and 3'-sides of the *dec* gene respectively. This PCR fragment was cloned into pUC19 EcoRI/SalI.and *E. coli* cells transformed with the resulting pUC19/T5Δ*dec* plasmid were infected with T5wt. The phage progeny were titrated to individual plaques, which were transferred to Hybond-N membranes (Amersham) and hybridized with a ^{32}P-labeled oligonucleotide covering the opposite sides of the deleted region (5'-GTAGCTCCTGAAGCATCATTCTTAATCTCCCT-3'). Deleted mutants were screened by hybridization overnight at room temperature in 2xSSC, 0.1% SDS, 0.1 mg/mL calf thymus DNA, followed by incubation for 30 min in 2xSSC, 0.1% SDS at 65°C and autography with PhosphorImager (Cyclone, Packard) and OptiQuant software. Positive plaques were selected as desired T5Δ*dec* mutant phages and checked by sequencing.

In order to produce non-decorated T5 heads, we constructed the double mutant T5*D18*am-Δ*dec* by cross-infection of the *E. coli* CR63 suppressive strain with T5Δ*dec* and T5*D18*amH5 mutant defective in tail assembly[13]. Phage progeny were plated on suppressive CR63 and non-suppressive F strains. Individual plaques exhibiting the amber phenotype were screened for the presence or absence of pb10 production by dot blots using anti-pb10 antibodies.

Capsid concentration determination. The empty capsids molar concentrations C_{mol} were determined based on the OD_{280} and OD_{260} as follows:

$$C_{mol} = OD_{280} \text{ x } \frac{F \text{ x } R_{th} \text{ x } (R_{exp} - R)}{MW_C \text{ x } E \text{ x } (1 - R_{th} \text{ x } R)}$$

where F = 1.47 (correction factor based on the amino acids quantitative analysis), R_{th} = 1.58 (theoretical OD_{260}/OD_{280} ratio based on the absorbance of the major head protein aromatic amino acids), R_{exp} is the measured OD_{260}/OD_{280} ratio, R = $280^4/260^4$, MW_C = 26 MDa (capsid molecular weight), E = $\varepsilon_{pb8}/MW_{pb8}$ = 0.878 (ε_{pb8} is the major head protein extinction coefficient and MW_{pb8} is the major head protein molecular weight).

Anti-pb10 and anti-capsid antibodies. Rabbit polyclonal antisera raised against pb10 and empty capsids were produced according to standard protocols [14], and IgG fractions were purified by affinity chromatography using HiTrap protein A columns as recommended by the supplier (GE Healthcare).

Negative stain imaging of T5 particles. A 3 μL drop of the sample was deposited onto a formvar/carbon film 300 mesh grid (EMS, Hatfield, USA) previously treated by plasma glow discharge. The sample was then contrasted by negative staining with 1% uranyl acetate. The specimen was observed with a JEM 2010 field emission gun transmission electron microscope (JEOL, Tokyo, Japan) using an acceleration voltage of 200 kV. Images were acquired with a Gatan 4K x 4K Ultrascan camera (Gatan, Pleasanton, USA).

Supplementary references

1. Ashkenazy, H. *et al.* ConSurf 2016: an improved methodology to estimate and visualize evolutionary conservation in macromolecules. *Nucleic Acids Res.* gkw408 (2016). doi:10.1093/nar/gkw408
2. Robert, X. & Gouet, P. Deciphering key features in protein structures with the new ENDscript server. *Nucleic Acids Res.* **42,** W320–W324 (2014).
3. Svergun, D., Barberato, C. & Koch, M. H. J. CRYSOL – a Program to Evaluate X-ray Solution Scattering of Biological Macromolecules from Atomic Coordinates. *J. Appl. Crystallogr.* **28,** 768–773 (1995).
4. Svergun, D. I., Petoukhov, M. V. & Koch, M. H. Determination of domain structure of proteins from X-ray solution scattering. *Biophys. J.* **80,** 2946–2953 (2001).
5. Palmer, A. G., Rance, M. & Wright, P. E. Intramolecular motions of a zinc finger DNA-binding domain from Xfin characterized by proton-detected natural abundance carbon-13 heteronuclear NMR spectroscopy. *J. Am. Chem. Soc.* **113,** 4371–4380 (1991).
6. Whitmore, L. & Wallace, B. A. DICHROWEB, an online server for protein secondary structure analyses from circular dichroism spectroscopic data. *Nucleic Acids Res.* **32,** W668–W673 (2004).
7. Micsonai, A. *et al.* Accurate secondary structure prediction and fold recognition for circular dichroism spectroscopy. *Proc. Natl. Acad. Sci.* **112,** E3095–E3103 (2015).
8. Brooks, B. R. *et al.* CHARMM: A program for macromolecular energy, minimization, and dynamics calculations. *J. Comput. Chem.* **4,** 187–217 (1983).
9. Laskowski, R. A., MacArthur, M. W., Moss, D. S. & Thornton, J. M. PROCHECK: a program to check the stereochemical quality of protein structures. *J. Appl. Crystallogr.* **26,** 283–291 (1993).
10. Laskowski, R. A., Rullmann, J. A. C., MacArthur, M. W., Kaptein, R. & Thornton, J. M. AQUA and PROCHECK-NMR: Programs for checking the quality of protein structures solved by NMR. *J. Biomol. NMR* **8,** 477–486 (1996).
11. Konarev, P. V., Volkov, V. V., Sokolova, A. V., Koch, M. H. J. & Svergun, D. I. PRIMUS: a Windows PC-based system for small-angle scattering data analysis. *J. Appl. Crystallogr.* **36,** 1277–1282 (2003).
12. Volkov, V. V., Svergun, D. I. & IUCr. Uniqueness of ab initio shape determination in small-angle scattering. *J. Appl. Crystallogr.* (2003).
13. Hendrickson, H. E. & McCorquodale, D. J. Genetic and physiological studies of bacteriophage t5 I. An expanded genetic map of t5. *J. Virol.* **7,** 612–618 (1971).
14. Harlow, E. & Lane, D. *Antibodies: a laboratory manual*. (1988).
